# Effects of High-Dextrose Intake on the Structural and Functional Properties of the Small Intestine in Young-Maturing and Middle-Aged Rats

**DOI:** 10.3390/ijms27167176

**Published:** 2026-08-11

**Authors:** Anđelija Gudelj, Ilijana Grigorov, Vesna Otašević, Nevena Savić, Milica Markelić, Ana Stančić, Nikola Dajić, Ksenija Veličković

**Affiliations:** 1 Department of Molecular Biology, Institute for Biological Research “Siniša Stanković”, National Institute of Republic of Serbia, University of Belgrade, 11000 Belgrade, Serbia; 2 Department of Cell and Tissue Biology, Faculty of Biology, University of Belgrade, 11000 Belgrade, Serbia

**Keywords:** dextrose overload, small intestine, intestinal barrier integrity, epithelial turnover kinetics, oxidative stress, redox-sensitive proteins, HMGB1/NF-κB p65/Nrf2 signalling, inflammation

## Abstract

Excessive dietary dextrose intake and its misuse in sports raise significant concerns about health outcomes across life stages. To investigate how biological maturity influences small intestinal responsiveness to dextrose overexposure, we subjected 1-month-old (young maturing) and 14-month-old (middle-aged) male Wistar rats to a 20% or 60% dextrose drinking regimen for 8 weeks. The middle-aged rats receiving the 60% dextrose solution exhibited the most pronounced alterations, including compromised barrier integrity, reduced crypt proliferation, and accelerated enterocyte apoptosis and necrosis. These structural changes coincided with weakened antioxidant defences, tissue iron accumulation, and elevated lipid peroxidation. Altered neuroendocrine and purinergic pathways, marked by increased serotonin, neuron-specific enolase (NSE), cytosolic high mobility group box 1 (HMGB1), and ATP synthase, alongside downregulated P2X7 receptor expression and a reduced nuclear factor kappa B p65/nuclear factor erythroid 2-related factor 2 (NF-κB p65/Nrf2) ratio, indicate potential observational associations with metabolic exhaustion and impaired inflammatory responsiveness under a chronic high-dextrose regimen. In conclusion, while fasting homeostatic model assessment of insulin resistance (HOMA-IR) values remained stable, a high-dextrose drinking regimen promotes maturity-dependent small intestinal changes and molecular signalling weakness, providing a preliminary pathophysiological framework for understanding potential health outcomes of dextrose overexposure across different life stages.

## 1. Introduction

The significant rise in sugar intake, particularly refined sugars, is a worldwide health concern and it is associated with a high risk of metabolic syndrome, type 2 diabetes, obesity, cardiovascular disease and cancers [1]. According to the World Health Organization (WHO), adults and children should reduce their daily intake of free sugars (refined and added) to less than 10% of total energy intake because of their negative effects on non-communicable diseases, including those affecting the intestinal and immune systems [2]. Dextrose, a refined sweetener derived from starch hydrolysis, is among the most prevalent ingredients in the modern food industry. Chemically, dextrose is identical to D-glucose, the primary endogenous monosaccharide in blood circulation, and the terms are often used interchangeably. However, in this study, the term “dextrose” is used specifically to distinguish this exogenous dietary additive from endogenous blood glucose and the total basal carbohydrate load. Because of its rapid absorption, dextrose is also used clinically to treat hypoglycaemia, manage acute alcohol intoxication, and assist endurance athletes in preventing glycogen depletion [3].

Physiologically, glucose is rapidly absorbed in the small intestine at low concentrations via sodium–glucose linked transporters (SGLT1), followed by facilitated diffusion mediated by glucose transporter 2 (GLUT2) located in the basolateral membrane [4]. At high luminal glucose concentrations, the abundance of SGLT1 on the apical surface of enterocytes increases, and glucose flux from the intestinal lumen to the apical membrane activates protein kinase C signalling. This activation induces rapid translocation of GLUT2 to the apical membrane, increasing glucose transport [4,5]. These events are specific adaptations of the small intestine, developed to meet the varying energetic demands of monosaccharide absorption. In non-specific adaptations of the small intestine, the total absorptive capacity, or absorptive surface area, changes through alterations in the length of the small intestine, the height of the villi, the length of the microvilli, and the number, size, and degree of differentiation of enterocytes [6].

While epidemiological data clearly link excessive sugar consumption to dysmetabolism and systemic oxidative stress, the precise impact of dietary sugars on gastrointestinal structure and function remains poorly understood. Beyond acting as a physical barrier, the intestinal epithelium is a highly metabolic, dynamic tissue responsible for nutrient absorption, immune defence, hormone secretion and continuous self-renewal, processes that render it uniquely susceptible to oxidative damage. At the intestinal level, high-sugar intake can compromise morphology, alter the microbiota profile and increase permeability, although these outcomes vary with sugar type, dosage, exposure time and animal age [7,8]. Notably, high-glycaemic index sugars such as dextrose are strongly associated with elevated oxidative stress [9]. This vulnerability is further compounded in the proximal small intestine, the primary site of iron absorption. Simple sugars can chelate inorganic iron into stable, low-molecular-weight, soluble complexes, facilitating its transport across the mucosa [10,11]. Consequently, high-sugar diets, particularly those rich in fructose, increase non-haem iron bioavailability, potentially driving iron overload in both the liver and the intestinal mucosa. This accumulation triggers reactive oxygen species (ROS) production via the Fenton reaction, leading to mucosal oxidative stress, tight junction disruption, lipid peroxidation and localised inflammation [12]. Unlike the well-characterized pathways of fructose-induced pathology, systematic evaluations and reviews examining the impact of glucose consumption on intestinal iron absorption report heterogeneous and conflicting findings, ranging from negligible effects to significantly altered mucosal uptake [13,14]. These inconsistencies are primarily attributed to differences in experimental models, varying iron statuses of the hosts, and the specific duration of glucose exposure.

As the largest host–environment interface, the intestinal tract relies on a complex barrier system comprising mucus, epithelial cells, tight junctions and the microbiome to prevent the entry of toxins and pathogens [15]. However, dietary factors can disrupt this barrier, increasing permeability and triggering chronic low-grade inflammation, driven in part by downstream activation of the nuclear factor kappa B (NF-κB) p65 transcription factor and cytokine upregulation [1,7,16]. This barrier disruption, together with oxidative stress, can induce both passive and active release of high-mobility group box 1 (HMGB1) protein. Beyond its intranuclear and cytoplasmic roles as a DNA chaperone and regulator of cell death, extracellular HMGB1 is the prototypical damage-associated molecular pattern (DAMP) molecule, playing a critical role in signalling across multiple biological processes [17,18]. It can exacerbate barrier damage by activating NF-κB p65 via Toll-like receptor 4 (TLR4) [19] and can further impair tight junctions through the receptor for advanced glycation end-products (RAGE) and extracellular signal-regulated kinase (ERK1/2) pathway [19]. Furthermore, recent evidence indicates that HMGB1 induces glutathione peroxidase 4 (GPX4)-mediated ferroptosis via TLR4/NF-κB p65 [20], identifying it as a critical mediator of intestinal pathology across diverse aetiologies.

Nuclear factor erythroid 2-related factor 2 (Nrf2) is a key regulator of the cellular defense against oxidative stress and inflammation, which plays a crucial role in maintaining gastrointestinal homeostasis [21,22]. Beyond upregulating antioxidant enzymes, Nrf2 alleviates intestinal pathology by suppressing pro-inflammatory pathways, specifically through the downregulation of NF-κB p65 and the inhibition of HMGB1 release [23,24,25]. Conversely, NF-κB p65 can reciprocally modulate Nrf2 activity, underlining a complex, cell- and stressor-dependent crosstalk between these two redox-sensitive transcription factors [26]. Despite the well-established interplay within the HMGB1/NF-κB p65/Nrf2 pathways, its specific dynamics in the small intestine under conditions of elevated sugar consumption, particularly dextrose, remain completely unexplored.

The structural and functional plasticity of the small intestine changes significantly throughout life. Despite the widespread consumption of refined sugars, it remains unclear how biological maturity affects tissue vulnerability to chronic dextrose overexposure. In this study, we used a liquid dextrose model to simulate real-world scenarios. Young, 1-month-old (maturing) and 14-month-old (middle-aged adult) male Wistar rats were subjected to an eight-week drinking regimen containing either a 20% or 60% dextrose solution. This setup reflects heavy soft drink consumption during rapid juvenile development, as well as dense glucose supplementation in middle-aged individuals. The use of the 60% dextrose solution was chosen as a physiological challenge to deliberately saturate active epithelial transport systems. In a real-world human context, this corresponds to the use of hypercaloric mass gainers or concentrated carbohydrate-loading products, where sugar densities routinely reach 40% to 70%. From a physiological standpoint, this approach allowed us to examine how dextrose overexposure affects small intestinal barrier regulation at metabolic saturation. We evaluated small intestinal changes by analysing tissue morphology, epithelial cell turnover kinetics, and tight junction integrity. Additionally, we examined local neuroendocrine and purinergic signalling, glucose transport, bioenergetic characteristics, tissue iron accumulation, antioxidant enzyme activity, and the subcellular redistribution of the redox-sensitive transcription factors HMGB1, NF-κB p65, and Nrf2. Focusing on these specific life stages enables a better understanding of the potential health consequences of excessive dextrose intake and the use of sports supplements across different ages.

## 2. Results

### 2.1. Energy Intake and Glucose Metabolism in Young Maturing and Middle-Aged Adult Rats Following a High-Dextrose Drinking Regimen

To evaluate the effects of the eight-week dextrose intervention in young maturing and middle-aged adult rats, systemic metabolic parameters, body weight, and food and liquid intake were measured, allowing precise calculation of daily caloric and dextrose consumption profiles (Table 1). Two-way ANOVA showed a significant main effect of maturity on body weight (*p* < 0.001), while Tukey’s post hoc test revealed no significant changes over time within the older, middle-aged groups. However, total weight gain was significantly influenced by maturity (*p* < 0.001), dextrose regimen (*p* < 0.05), and their interaction (*p* < 0.001). Post hoc multiple comparisons demonstrated a significant increase in weight gain in the 20D young maturing and middle-aged adult rats, as well as in the 60D middle-aged group, compared with their respective untreated controls (*p* < 0.05 and *p* < 0.001, respectively). Daily food consumption analysed at the cage level using independent values normalised per animal to account for different housing densities (3 young maturing vs. 2–3 middle-aged rats per cage) was significantly altered by the dextrose regimen (*p* < 0.001), with a significant interaction between the two variables (*p* < 0.01) on the reduction in food intake. A statistically significant difference was observed in 20D and 60D young maturing rats compared with the appropriate controls (*p* < 0.01 and *p* < 0.05, respectively), as well as in dextrose-treated middle-aged adults compared with age-matched controls (*p* < 0.001 for both). Liquid intake was independently influenced by maturity (*p* < 0.01) and dextrose regimen (*p* < 0.001), with a significant interaction (*p* < 0.05). The most pronounced statistically significant changes occurred in the 20D middle-aged adult rats compared with the corresponding control group (*p* < 0.001), the 20D young maturing rats (*p* < 0.01), and the 60D middle-aged rats (*p* < 0.05). Although total caloric intake showed an increasing trend in groups on the high-dextrose drinking regimen without reaching statistical significance, the proportion of total caloric intake derived solely from dextrose was significantly higher in the 20D and 60D middle-aged adult rats than in the younger rats under the same feeding conditions (*p* < 0.05 and *p* < 0.01, respectively). The resulting HOMA-IR index ranged from 0.915 to 1.241, confirming that normal insulin sensitivity was maintained throughout the intervention. (Table 1).

**Figure 1:**
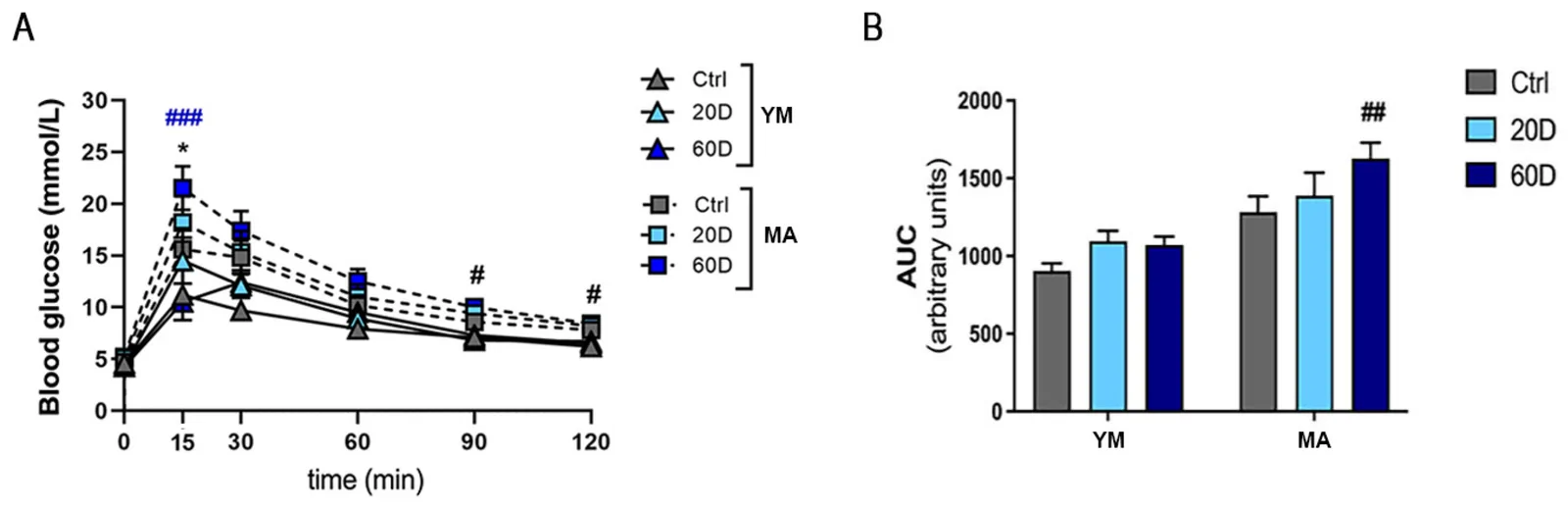
Intraperitoneal glucose tolerance test (IP-GTT) and blood glucose kinetics in young maturing (YM) and middle-aged (MA) adult rats following a high-dextrose drinking regimen. (**A**) Glucose tolerance curve monitored for 120 min after glucose administration in young maturing and middle-aged adult rats in control (Ctrl), 20% dextrose (20D), and 60% dextrose (60D) groups. (**B**) Total area under the curve (AUC) expressed in arbitrary units. Values are expressed as mean ± SEM (n = 9 independent biological replicates per group). Statistical evaluations for the blood glucose kinetics curve (**A**) were performed using two-way repeated-measures ANOVA followed by Tukey’s post hoc test, with Greenhouse–Geisser corrections applied where appropriate. Statistical evaluations for the total AUC values (**B**) were performed using independent-measures two-way ANOVA followed by Tukey’s post hoc test. Comparison of treated groups with the corresponding control group (*); comparison of rats at different stages of maturity under the same dextrose drinking conditions (#). Statistical significance was set at *p* < 0.05, with *, # *p* < 0.05; ## *p* < 0.01; ###, *p* < 0.001.

To assess glucose homoeostasis and potential glucose intolerance, an intraperitoneal glucose tolerance test (IP-GTT) was performed. As shown in Figure 1A, blood glucose levels peaked (*p* < 0.001) in the 60D middle-aged adult rats compared with the 60D young maturing rats and remained elevated after 15 min. A statistically significant difference in blood glucose levels was also observed after 15 min between the non-treated control groups (*p* < 0.05). Furthermore, a significantly higher glucose response was sustained at 90 and 120 min (both *p* < 0.05) in the 60D middle-aged adult rats compared with the 60D younger animals. Two-way repeated-measures ANOVA revealed significant main effects of maturity (*p* < 0.001) and dextrose regimen (*p* < 0.001), as well as a significant interaction effect (*p* < 0.001) across the IP-GTT kinetic curve. These physiological differences were further confirmed by analysing the total area under the curve (AUC) of the IP-GTT (Figure 1B). Two-way ANOVA for the total AUC data showed significant main effects of both maturity (*p* < 0.001) and dextrose regimen (*p* < 0.05), with no significant interaction between the two variables. Specifically, post hoc analysis demonstrated that the IP-GTT AUC in the 60D middle-aged adult rats was significantly higher than in their younger counterparts (*p* < 0.01), indicating an early overall impairment in systemic glucose handling under a chronic high-dextrose regimen (Table 1).

### 2.2. Dose- and Age-Dependent Effects of a High-Dextrose Drinking Regimen on Small Intestine Morphology and Function

**Figure 2:**
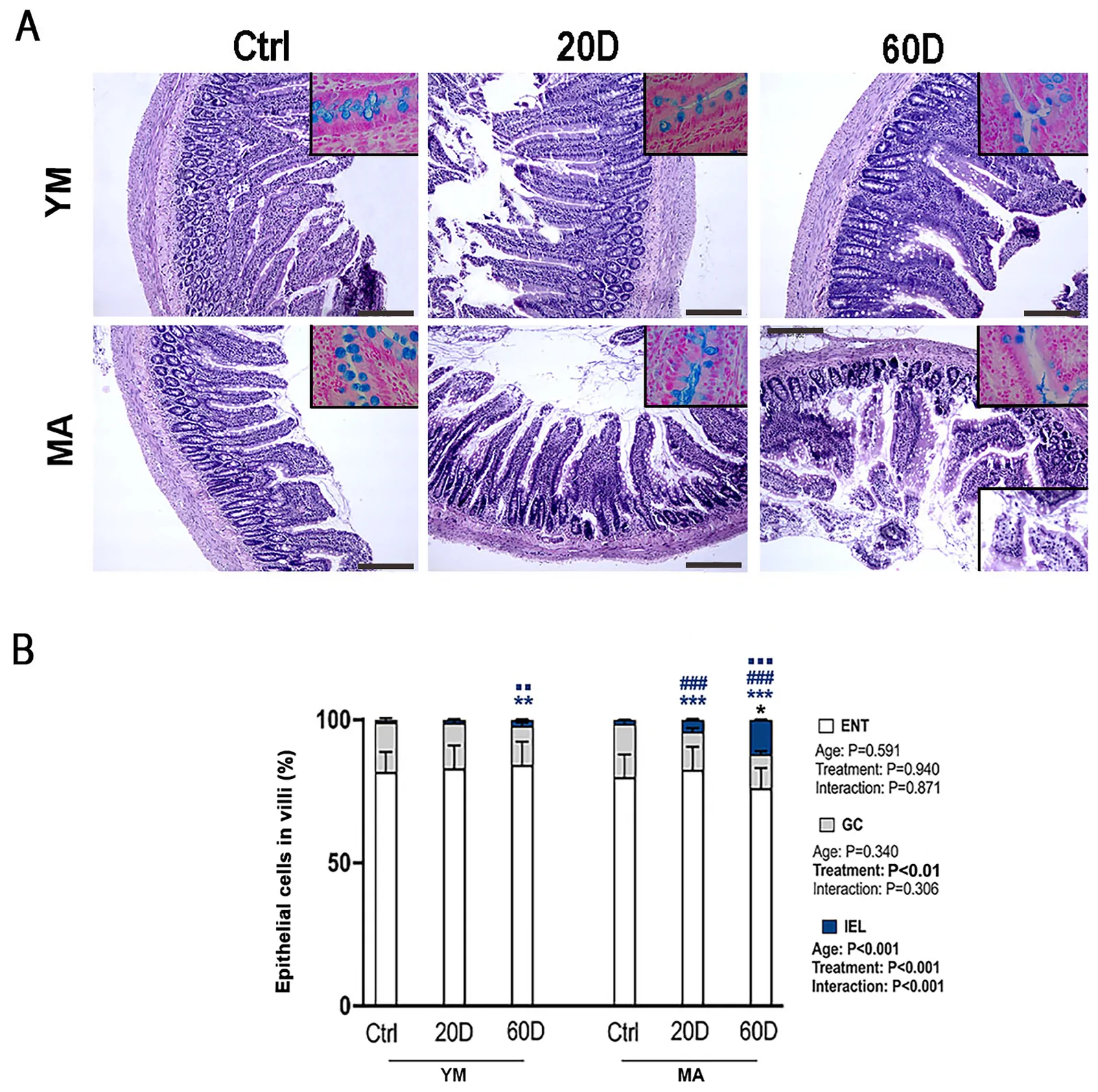
Histomorphological distribution of villus epithelial cell subpopulations in the small intestine of young maturing (YM) and middle-aged (MA) adult rats following a high-dextrose drinking regimen. (**A**) Representative histological micrographs (H&E) of the small intestinal mucosa in young maturing and middle-aged adult rats under control (Ctrl), 20% dextrose (20D), and 60% dextrose (60D) regimens. Enlarged detail (bottom inset) shows mononuclear infiltrate in the lamina propria, frequent intestinal cell death, and vacuolization in the middle-aged group treated with 60% dextrose. Alcian blue staining indicates mucins within the goblet cells (GCs) and the secreted mucus layer (upper insets); ×10 scale bars: 200 µm. (**B**) Quantitative proportional distribution of epithelial cell subpopulations (%) within the intestinal villi, categorized as enterocytes (ENT), goblet cells (GCs), and intraepithelial lymphocytes (IELs). Statistical analyses were performed using independent-measures two-way ANOVA followed by Tukey’s post hoc test, with the resulting *p*-values for the main two-way ANOVA factors shown adjacent to the graph separately for each cell lineage. Values are expressed as mean ± SEM (n = 3 independent biological replicates per group), with each value derived from 10 non-overlapping microscopic technical fields per animal to prevent pseudoreplication. Comparison of dextrose-treated groups with the corresponding control group (*); comparison of rats at different stages of maturity under the same dextrose drinking conditions (#); comparison of rats at the same maturity stage under different dextrose drinking regimens (▪). Statistical significance was set at *p* < 0.05, with * *p* < 0.05; **, ▪▪ *p* < 0.01; ***, ###, ▪▪▪ *p* < 0.001.

Morphological changes related to both maturity and dextrose regimen between the experimental groups are shown in Figure 2A. Histological screening of samples from young maturing control rats shows a typical small intestinal architecture. The intestinal wall displayed long villi covered by columnar epithelium with firmly adherent enterocytes and goblet cells. Moderate numbers of intraepithelial lymphocytes (IELs) were also noted. The villi are adjacent to the intestinal crypts at their base and are surrounded by the lamina propria. In the lamina propria of the villus core, mainly mononuclear cells and normally structured lymphatic vessels (lacteals) were observed. Normal histological structure was also seen in the submucosa and muscle layer of both young maturing and middle-aged adult control rats. Both young maturing and middle-aged adult rats on the 20% dextrose drinking regimen showed a similar histological structure to the corresponding control rats. Microscopic evaluation of the small intestine wall in middle-aged adult rats showed villus shortening, numerous IELs, abundant infiltration of the lamina propria with mononuclear cells, disruption of the epithelium, and partial loss of the brush border. Compared to 60D young maturing rats, these changes were more pronounced in 60D middle-aged adult rats. Histological observation revealed that the number of mucin-producing goblet cells (GCs) decreased in the 60D middle-aged adult group compared to the other experimental groups. In addition, numerous cells undergoing cell death were observed in the 60D middle-aged adult rats. Some cells showed morphological features of apoptosis, such as pyknosis and dark cytoplasm, while others exhibited signs of necrosis, including cytoplasmic swelling and vacuolisation. In the group of 60D middle-aged adult rats, many cells in the upper third of the intestinal villi showed necrotic alterations.

Next, we investigated whether changes in intestinal morphology were associated with shifts in the proportions of epithelial cell subpopulations within the villi (Figure 2B). We observed no statistically significant main effects of maturity, dextrose regimen, or their interaction on the proportion of enterocytes (ENT) across the experimental groups. However, there was a descriptive trend towards a reduction in the proportion of ENT in middle-aged adult rats compared with young maturing rats. Another key feature of the intestinal epithelium is the presence of the previously mentioned GCs, which constitute the primary line of defence in the gastrointestinal tract, and IELs, a population of immune cells located closest to the luminal surface between adjacent epithelial cells. Two-way ANOVA showed a significant main effect of the high-dextrose drinking regimen on the proportion of GCs (*p* < 0.01). A significant main effect of maturity was also found on the proportion of IELs (*p* < 0.001), alongside an independent effect of dextrose regimen (*p* < 0.001) and a significant interaction effect between maturity and dextrose regimen (*p* < 0.001), which returned the proportion to the control level. The most pronounced changes in both GCs and IELs were documented in the 60D middle-aged adult group, suggesting a compromised GC profile and ongoing inflammatory strain within the small intestinal wall.

The microscopic observations were further evaluated via morphometric analysis (Table 2). Our analysis focused on the following structural features: villus height and width, crypt depth, muscle layer thickness, and villus surface area (VSA), which tracks potential adaptive changes in the absorptive surface under dextrose overexposure. Two-way ANOVA showed a significant main effect of maturity (*p* < 0.05) on villus height, although Tukey’s post-hoc multiple comparison test revealed no statistical differences between the specific experimental groups. There was also an individual main effect of maturity on the reduction in crypt depth (*p* < 0.05). Furthermore, two-way ANOVA revealed a significant main effect of maturity (*p* < 0.01) alongside a significant interaction effect between maturity and dextrose regimen (*p* < 0.05) on VSA. Tukey’s post-hoc analysis demonstrated that the middle-aged adult rats maintained an increased overall VSA compared to their young counterparts (*p* < 0.01). Finally, a significant main effect of maturity was observed on muscle layer thickness (*p* < 0.05), with the lowest descriptive values found in the 60D middle-aged adult rats. However, Tukey’s post-hoc test showed no statistically significant differences between individual groups (Table 2).

### 2.3. Epithelial Cell Turnover in Young Maturing and Middle-Aged Adult Rats Following a High-Dextrose Drinking Regimen

**Figure 3:**
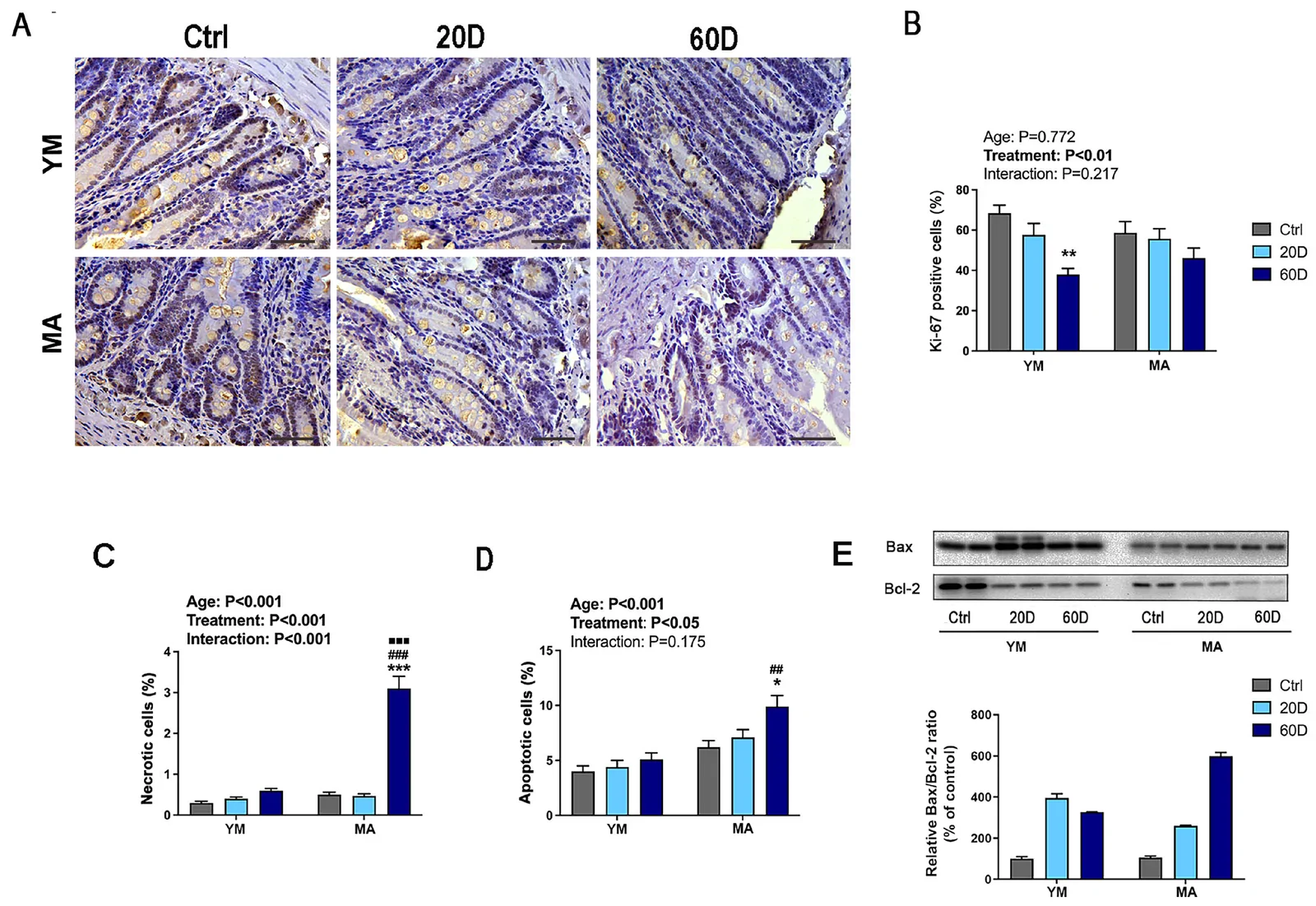
Evaluation of intestinal epithelial cell turnover via proliferation, apoptosis, and necrosis in young maturing (YM) and middle-aged (MA) adult rats following a high-dextrose drinking regimen. (**A**) Representative immunohistochemical micrographs showing Ki-67 immunopositivity within the crypts of the small intestine in young maturing and middle-aged adult rats in control (Ctrl), 20% dextrose (20D), and 60% dextrose (60D) groups. (**B**) Quantitative analysis of Ki-67-positive cells (%), indexing the rate of epithelial cell proliferation across maturity groups and dextrose regimens. Two-way ANOVA (*p*) values for the main factors are shown above the respective bar graphs; ×40; scale bar: 50 µm. (**E**) Representative Western blot for Bax and Bcl-2 alongside the descriptive densitometric quantification of the relative Bax/Bcl-2 ratio, expressed as a percentage of the young maturing control group (standardized to 100%). Values are expressed as mean ± SEM. Statistical evaluations for the quantitative imaging protocols (**B**–**D**) were performed using independent-measures two-way ANOVA followed by Tukey’s post hoc test (n = 3 independent biological replicates per group, with each value derived from 10 non-overlapping technical microscopic fields per animal). Densitometric quantification in panel (**E**) was utilized strictly for the descriptive evaluation of trends from pooled tissue specimens (n = 2 independent analytical lanes per group, with each lane comprising pooled homogenates from 4 animals) and was excluded from inferential statistical models due to sample pooling constraints. Comparison of treated groups with the corresponding control group (*); comparison of rats at different stages of maturity under the same dextrose drinking conditions (#); comparison of rats at the same maturity stage under different dextrose drinking regimens (▪). Statistical significance was set at *p* < 0.05, with * *p* < 0.05; **, ## *p* < 0.01; ***, ###, ▪▪▪ *p* < 0.001.

The growth and function of the small intestine are closely linked to the rapid turnover of epithelial cells, which is determined by the balance between cell proliferation and cell death. To clarify proliferation kinetics, we performed immunohistochemical screening for Ki-67, a proliferation marker. As shown in Figure 3A, Ki-67-positive immunoreactivity was localised mainly within the intestinal crypts, with the highest positivity observed in the young maturing control group. Two-way ANOVA (Figure 3B) revealed a significant main effect of the high-dextrose drinking regimen on the percentage of Ki-67-positive nuclei (*p* < 0.01), with no significant main effect of maturity or interaction between the two factors. Post hoc comparisons demonstrated a significant reduction in the 60D young maturing group compared with its corresponding untreated control (*p* < 0.01).

Parallel epithelial cell loss was assessed based on the histomorphological features of apoptotic and necrotic enterocytes. For the proportion of necrotic cells (Figure 3C), significant independent effects of maturity (*p* < 0.001) and dextrose regimen (*p* < 0.001) were accompanied by a significant interaction effect (*p* < 0.001). A prominent increase in necrosis was documented in the 60D middle-aged adult group compared with its corresponding control (*p* < 0.001), the 60D young maturing rats (*p* < 0.001), and the 20D middle-aged adult rats (*p* < 0.001). Regarding the apoptotic indices, both maturity (*p* < 0.001) and dextrose regimen (*p* < 0.05) exerted independent, increasing main effects on apoptotic indices, without a significant interaction (Figure 3D). Post hoc analysis revealed a significant increase in enterocyte apoptosis in the 60D middle-aged adult rats compared with their age-matched controls (*p* < 0.05) and the young maturing control group (*p* < 0.01). Micrograph supporting these results is presented as Supplementary Figure S1. To further explore the presence of apoptosis, we descriptively assessed tissue levels of key apoptosis-regulating proteins (Figure 3E). Densitometric analysis of the relative Bax/Bcl-2 ratio, expressed as a percentage of the young maturing controls, revealed a strong descriptive trend towards an elevated pro-apoptotic signalling profile under the high-dextrose regimen. In line with our histomorphological apoptotic indices, the most pronounced increase in the Bax/Bcl-2 ratio was observed in the 60D middle-aged adult rats, reaching approximately 600% of control values. This corresponding molecular shift aligns with the observed acceleration of enterocyte cell death, suggesting that long-term dextrose consumption drives the middle-aged intestinal epithelium towards a pro-apoptotic state.

### 2.4. Dose- and Age-Dependent Effects of a High-Dextrose Drinking Regimen on Small Intestine Epithelial Barrier Integrity and Neuroendocrine Cell Function

**Figure 4:**
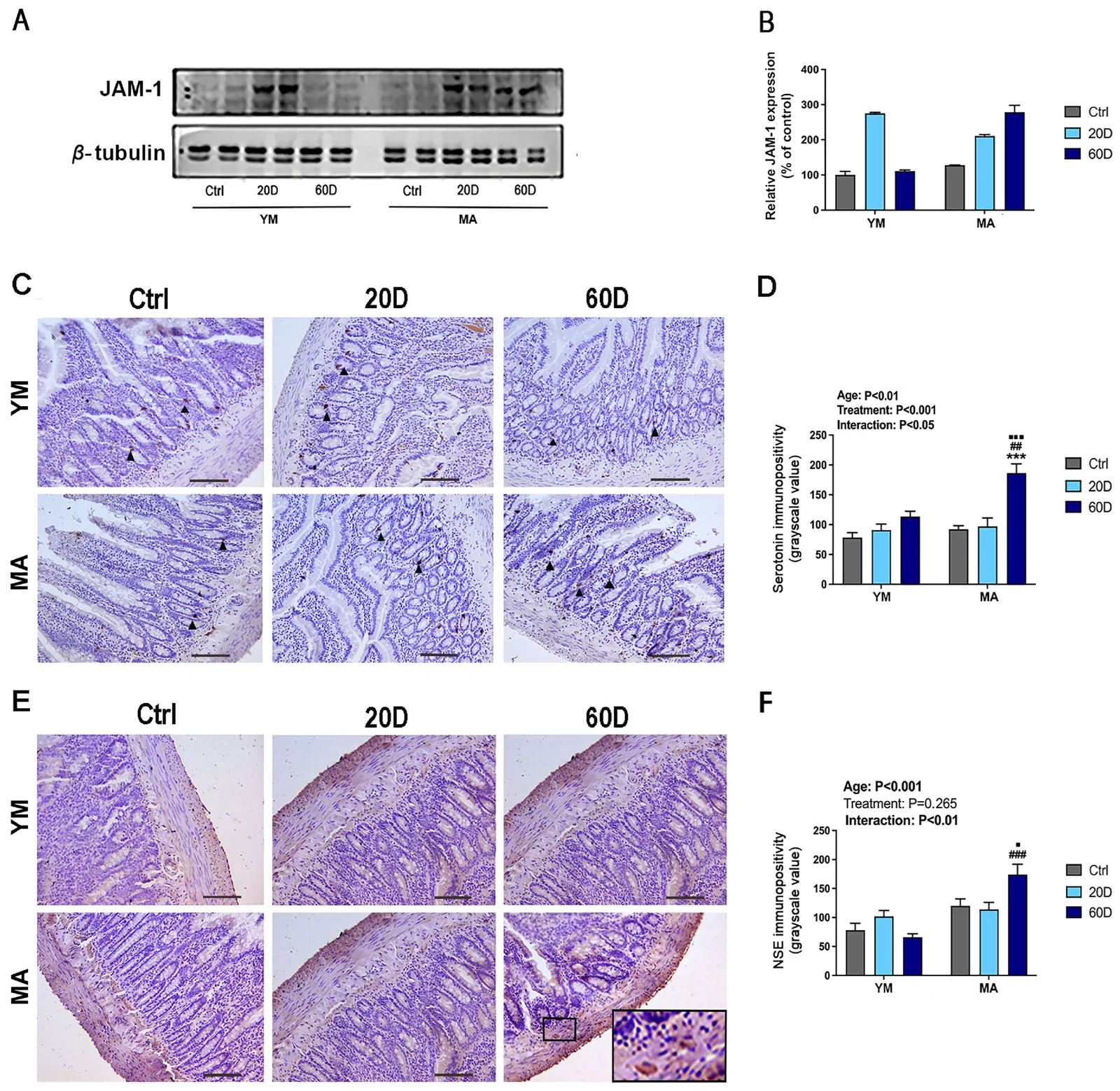
Assessment of junctional adhesion molecule 1 (JAM-1), serotonin, and neuron-specific enolase (NSE) expression in the small intestine of young-maturing (YM) and middle-aged (MA) adult rats following a high-dextrose drinking regimen (**A**) Representative Western blot analysis showing protein expression levels of JAM-1 and β-tubulin (loading control) in whole small intestine homogenate from young maturing and adult rats under control (Ctrl), 20% dextrose (20D), and 60% dextrose (60D) treatment conditions. (**B**) Descriptive densitometric trends (%) for JAM-1 protein expression in pooled tissue specimens, relative to young maturing controls (100%). No inferential statistical analysis was performed for Western blot panels because of sample pooling constraints. (**C**) Representative immunohistochemical micrographs showing serotonin localization and positivity within the small intestine tissue of young maturing and middle-aged adult rats. Black arrowheads highlight specific immunoreactive cells. (**D**) Quantitative analysis of serotonin immunopositivity, measured as mean grayscale values across all groups. (**E**) Representative immunohistochemical micrographs showing neuron-specific enolase (NSE) expression within the small intestine across experimental groups. The inset in the middle-aged 60D panel shows a higher-magnification view of NSE immunoreactivity in nerve plexuses. (**F**) Quantitative analysis of NSE immunopositivity, representing mean grayscale intensity values across maturity groups and dextrose drinking conditions, ×20; scale bar: 100 µm. Values are expressed as mean ± SEM. Statistical evaluations for immunohistochemistry quantification were performed using independent-measures two-way ANOVA followed by Tukey’s post hoc test, with the resulting *p*-values for maturity, treatment, and interaction factors indicated above the respective graphs (n = 3 independent biological replicates per group, with each value derived from 10 non-overlapping technical microscopic fields per animal). Comparison of treated groups with the corresponding control group (*); comparison of rats at different stages of maturity under the same dextrose drinking conditions (#); comparison of rats at the same maturity stage under different dextrose drinking regimens (▪). Statistical significance was set at *p* < 0.05, with ▪ *p* < 0.05; ## *p* < 0.01; ***, ###, ▪▪▪ *p* < 0.001.

Beyond absorption, the intestinal epithelium serves as a barrier that maintains integrity, regulates permeability, and supports immune balance. This function is mediated by tight junctions and protein complexes that control the passage of molecules between cells, with key integral membrane components including claudins, occludin, and junctional adhesion molecules (JAMs). JAM-1, a member of the immunoglobulin superfamily, is highly expressed in small intestinal epithelial cells and plays a crucial role in tight junction assembly and barrier regulation. To evaluate potential changes in mucosal barrier structural proteins, relative JAM-1 protein expression was assessed qualitatively by Western blot analysis across the experimental groups (Figure 4A). Densitometric analysis of the immunoblots (Figure 4B) revealed a maturity- and dose-dependent pattern of JAM-1 expression. In the young maturing rats, a pronounced increase in relative JAM-1 expression was observed primarily under the 20% dextrose regimen, whereas the 60% dextrose profile remained comparable to baseline control levels. In contrast, in middle-aged adult rats, 60% dextrose exposure produced a clear upward trend in relative JAM-1 expression compared with the respective control group and the corresponding younger rats. These qualitative trends suggest that host maturity influences the structural barrier response to different dextrose loads.

The small intestine functions as a complex sensory and endocrine organ, containing specialised epithelial enteroendocrine cells (EECs) that detect luminal stimuli, secrete bioactive hormones, and mediate communication between the gut and peripheral organs. Among these, enterochromaffin cells constitute a major EEC subtype responsible for serotonin production, a key mediator of gastrointestinal motility and signalling. To assess the presence and spatial distribution of serotonin within enterochromaffin cells, as well as neuron-specific enolase (NSE), a marker expressed in both neurons and neuroendocrine cells, immunohistochemical analysis was performed. Two-way ANOVA showed significant main effects of maturity (*p* < 0.01) and dextrose regimen (*p* < 0.001), as well as a significant interaction between maturity and dextrose regimen (*p* < 0.05). Post hoc analysis revealed a significant increase in serotonin-positive cells in the 60D middle-aged adult rats compared with their corresponding untreated control group (*p* < 0.001), the 60D young maturing rats (*p* < 0.01), and the 20D middle-aged adult rats (*p* < 0.001) (Figure 4C,D). Additionally, NSE immunoreactivity (Figure 4E) was observed mainly within the enteric nervous system (ENS), with the most pronounced response in the 60D middle-aged adult group. Localised NSE immunoreactivity was also detected in the lamina propria of the intestine in these 60D middle-aged rats, indicating the presence of NSE-positive nerve bundles. Two-way ANOVA revealed significant independent effects of maturity (*p* < 0.001) and a significant interaction between maturity and dextrose regimen (*p* < 0.01), with significantly higher values in the 60D middle-aged adult group compared with their young maturing counterparts and 20D middle-aged adult rats (*p* < 0.001 and *p* < 0.05, respectively, Figure 4F).

### 2.5. Dose- and Age-Dependent Effects of a High-Dextrose Drinking Regimen on GLUT2, P2X7, and ATP Synthase Expression and Localization in the Small Intestine

**Figure 5:**
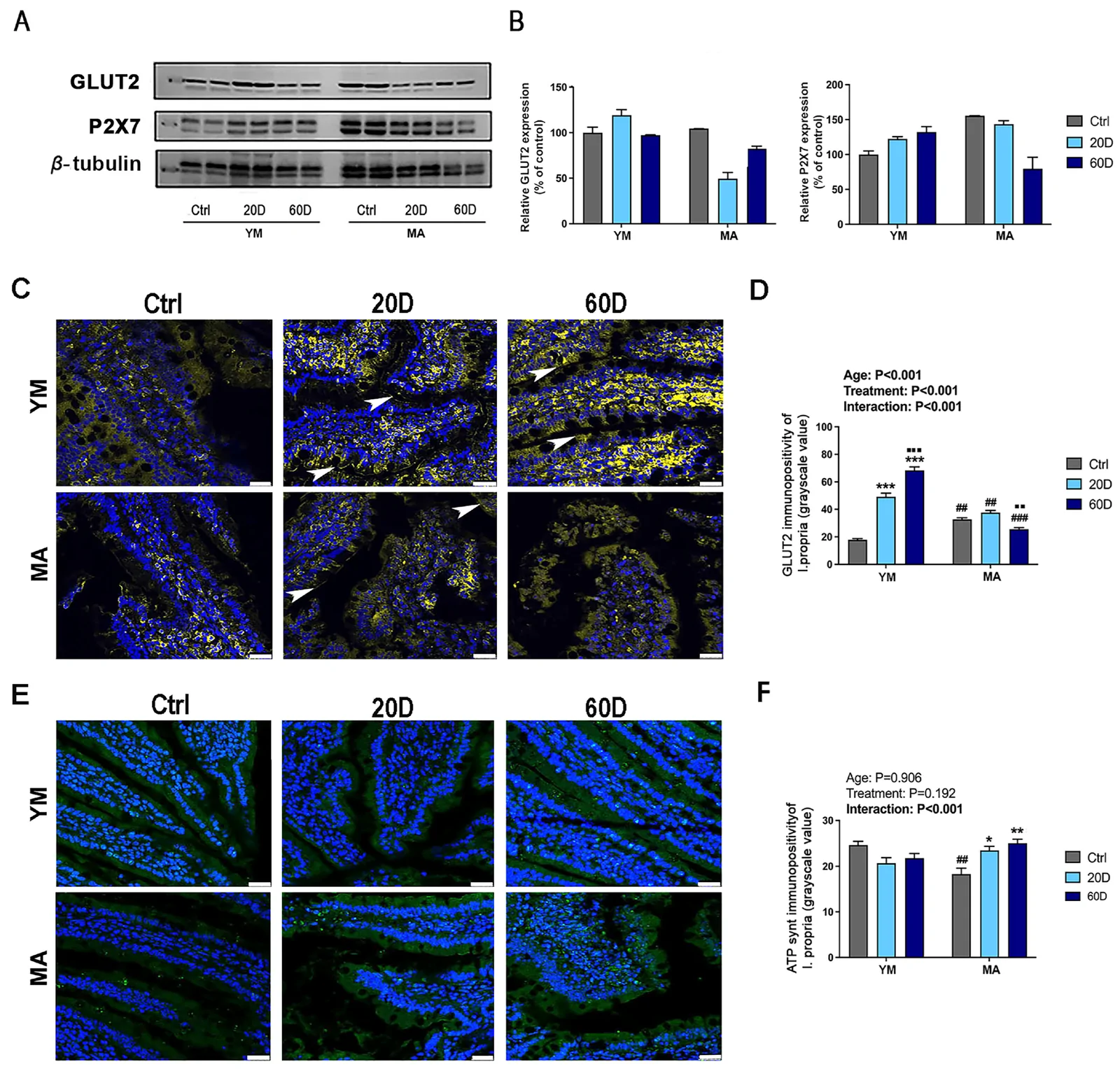
Assessment of glucose transporter 2 (GLUT2), P2X7 receptor, and ATP synthase expression and localisation in the small intestine of young-maturing (YM) and middle-aged (MA) adult rats following a high-dextrose drinking regimen. Representative Western blots (**A**) and descriptive densitometric trends (%) (**B**) for GLUT2 and P2X7 protein expression in pooled tissue specimens, relative to young maturing controls (100%). No inferential statistical analysis was performed for Western blot panels due to sample pooling constraints. (**C**,**D**) Representative immunofluorescence micrographs showing GLUT2 (yellow; arrows indicate apical localisation) counterstained with Sytox Orange (blue), and quantification of lamina propria greyscale intensity. (**E**,**F**) Representative immunofluorescence micrographs showing ATP synthase (green) counterstained with Sytox Orange (blue), and quantification of lamina propria greyscale intensity. Magnification ×63, scale bar: 25 µm. Values are expressed as mean ± SEM. Statistical evaluations for immunofluorescence quantification were performed using independent-measures two-way ANOVA followed by Tukey’s post hoc test, with the resulting *p*-values for maturity, dextrose treatment, and interaction factors indicated above the respective graphs (n = 3 independent biological replicates per group, with each value derived from 10 non-overlapping technical microscopic fields per animal). Comparison of treated groups with the corresponding control group (*); comparison of rats at different stages of maturity under the same dextrose drinking conditions (#); comparison of rats at the same maturity stage under different dextrose drinking regimens (▪). Statistical significance was set at *p* < 0.05, with * *p* < 0.05; **, ##, ▪▪ *p* < 0.01; ***, ###, ▪▪▪ *p* < 0.001.

Once inside the enterocyte, glucose moves down its concentration gradient via the facilitated diffusion transporter GLUT2 into the capillary blood for transport to the liver. To evaluate potential regulatory changes, GLUT2 and P2X7 protein expression profiles were examined using Western blot analysis, with densitometric data used strictly to present relative expression trends as a percentage of the respective controls (Figure 5). Densitometric evaluation of the immunoblots revealed a maturity- and dose-dependent pattern of GLUT2 expression (Figure 5A,B). Notably, young maturing and middle-aged adult rats exhibited completely opposite trends under the 20% dextrose regimen. In young maturing rats, the 20% dextrose drinking regimen induced an upward trend in relative GLUT2 expression compared with controls, whereas in middle-aged adults the same regimen caused a pronounced downward trend, representing the lowest expression levels across all groups. Following 60% dextrose overexposure, however, both groups showed a downward trend in relative GLUT2 expression compared with their respective 20% dextrose counterparts. In the young maturing rats, the 60D regimen brought the expression profile back down near baseline levels, while in the middle-aged adult rats, despite a slight upward shift from the low 20% point, the relative expression remained below middle-aged control levels. Because GLUT2 trafficking and localisation are tightly linked to the P2X7 receptor, P2X7 protein expression profiles were investigated in parallel. Densitometric evaluation indicated that within the control groups, the relative P2X7 signal was visibly higher in the middle-aged rats than in their younger counterparts. Following dextrose exposure, however, a marked decrease in the relative P2X7 profile was observed in the 60D middle-aged rats compared with the 60D young maturing rats, the middle-aged controls, and the middle-aged adult rats on the 20% dextrose regimen.

Quantification of the GLUT2 immunofluorescent signal in the lamina propria (Figure 5D) supported these microscopic observations. Two-way ANOVA revealed a significant main effect of dextrose regimen (*p* < 0.001), a significant main effect of maturity (*p* < 0.001), and a significant interaction effect (*p* < 0.001). The post hoc test showed a statistically significant increase in the young maturing group exposed to dextrose (20% and 60%) compared with the corresponding control group, and between 20D and 60D young maturing rats (*p* < 0.001) in all cases. A statistically significant decrease in immunopositivity was observed in the 60D middle-aged adult rats compared with their corresponding young counterparts and 20D middle-aged adult rats (*p* < 0.001 and *p* < 0.01, respectively). Furthermore, the decrease in GLUT2 immunopositivity in the 20D middle-aged adult group was statistically significant compared with the 20D young maturing rats (*p* < 0.01). Additionally, we investigated the immunoexpression of ATP synthase, as extracellular ATP activates the P2X7 receptor (Figure 5E,F). Two-way ANOVA showed a significant interactive effect of maturity and dextrose regimen on ATP synthase immunoexpression in the lamina propria (*p* < 0.001). Post hoc analysis revealed a significant decrease in middle-aged control rats compared with the corresponding young control group (*p* < 0.01). In contrast, a significant increase was observed in the 20D and 60D middle-aged adult rats compared with their respective untreated controls (*p* < 0.05 and *p* < 0.01, respectively).

### 2.6. Dose- and Age-Dependent Effects of a High-Dextrose Drinking Regimen on Small Intestinal Iron Deposition and Oxidative Stress Markers

**Figure 6:**
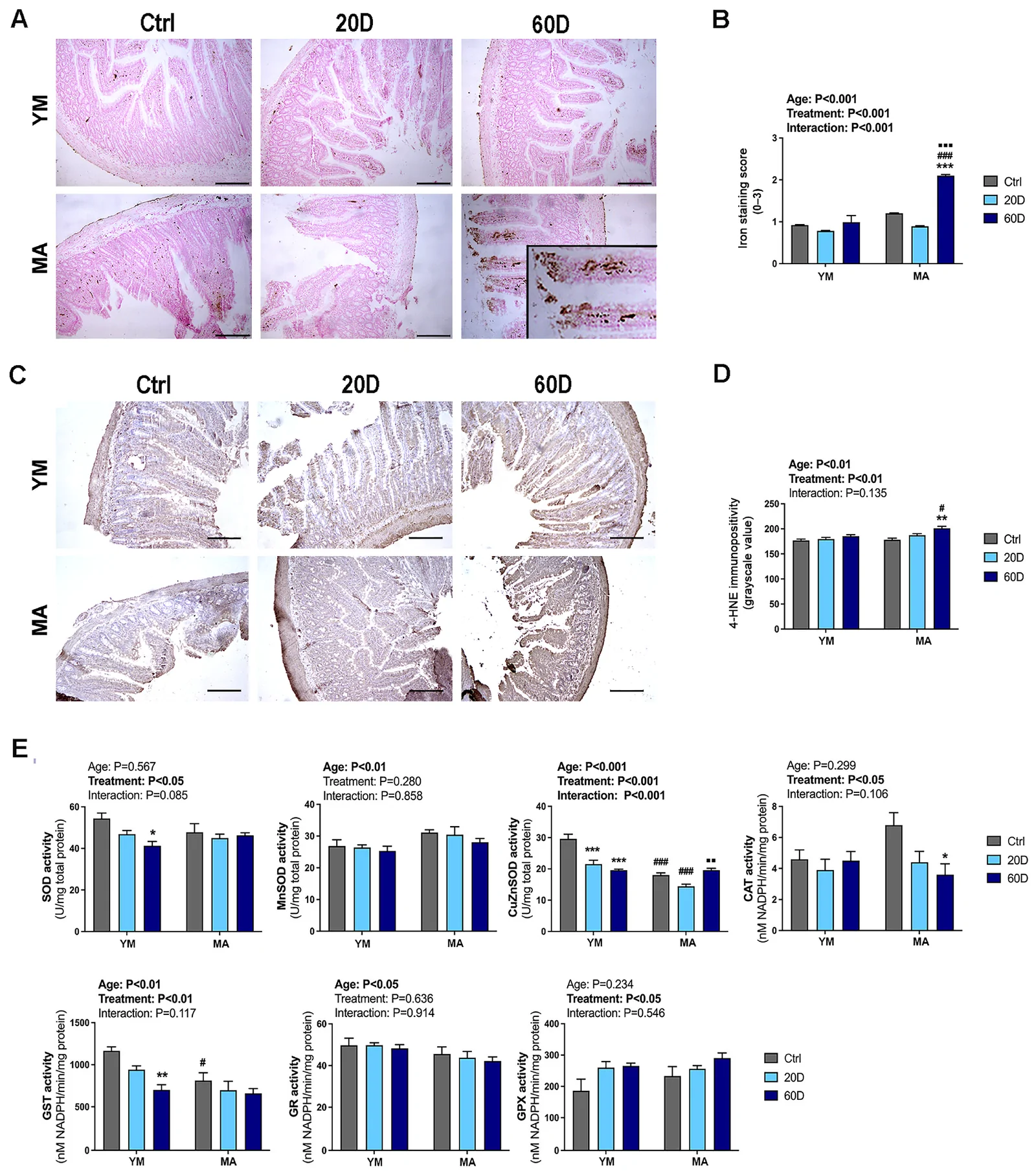
Dose- and age-dependent effects of a high-dextrose drinking regimen on small intestinal iron deposition and oxidative stress markers. (**A**) Representative histochemical micrographs showing Prussian blue detection of Fe^3+^ (indicated by brown staining) and regional iron distribution within the small intestine of young-maturing (YM) and middle-aged (MA) adult rats in control (Ctrl), 20% dextrose (20D), and 60% dextrose (60D) groups. The panel for 60D middle-aged adult rats includes an inset with enlarged details of Fe^3+^ deposition. Scale bar: 200 µm. (**B**) Semi-quantitative iron staining scores (0–3) assessing iron accumulation across the groups. (**C**) Representative immunohistochemical micrographs and (**D**) quantitative analysis of 4-hydroxynonenal (4-HNE) immunopositivity, measured as mean grayscale intensity values in the small intestine. × 10; scale bar: 200 µm. (**E**) Enzyme activities of total superoxide dismutase (SOD), manganese SOD (MnSOD), copper-zinc SOD (CuZnSOD), catalase (CAT), glutathione S-transferase (GST), glutathione reductase (GR), and glutathione peroxidase (GPX). Values are expressed as mean ± SEM. Statistical analyses were conducted using independent-measures two-way ANOVA, followed by Tukey’s post hoc test (n = 3 independent biological replicates per group for histochemistry and immunohistochemistry, obtained from 10 non-overlapping technical microscopic fields per animal; n = 9 independent biological replicates per group for enzyme activity assays). Comparison of treated groups with the corresponding control group (*); comparison of rats at different stages of maturity under the same dextrose drinking conditions (#); comparison of rats at the same maturity stage under different dextrose drinking regimens (▪). Statistical significance was set at *p* < 0.05, with *, #, *p* < 0.05; **, ▪▪ *p* < 0.01; ***, ###, ▪▪▪ *p* < 0.001.

A high-glucose environment in the small intestine can alter iron homeostasis. To investigate potential changes in iron metabolism, Fe^3+^ was detected histochemically in tissue sections using Prussian blue/DAB staining. As shown in Figure 6A, iron-containing cells were present in numerous erythrocytes across all groups, including both control groups. In addition to the erythrocytes, intracellular accumulation of free iron was observed in the epithelial cells of the villi, particularly at their tips, as well as in mononuclear cells in the lamina propria and within the enteric neuronal plexuses. Two-way ANOVA of the semi-quantitative iron scoring data (Figure 6B) showed that tissue iron level was significantly affected by maturity (*p* < 0.001), dextrose regimen (*p* < 0.001), and their interaction (*p* < 0.001). Post hoc testing demonstrated a significant increase in iron accumulation in the 60D middle-aged adult rats compared with their corresponding untreated control group, the 20D middle-aged adult rats, and the 60D young maturing rats (*p* < 0.001 for all comparisons).

Following the detection of increased tissue iron level, the accumulation of 4-hydroxynonenal (4-HNE), a major cytotoxic product of lipid peroxidation, was examined (Figure 6C). Whereas immunopositivity for 4-HNE was homogeneously distributed throughout the small intestine in control tissues, microscopic examination revealed numerous focal areas of 4-HNE accumulation and a stronger immunopositive reaction in the dextrose-exposed groups, particularly in the 60D middle-aged adult group. Two-way ANOVA (Figure 6D) showed that 4-HNE immunopositivity profiles were significantly affected by maturity (*p* < 0.01) and dextrose regimen (*p* < 0.01), without a significant interaction effect. A significant increase in 4-HNE immunopositivity was observed in the 60D middle-aged adult rats compared with their corresponding untreated control group and with young maturing rats maintained under the same drinking conditions (*p* < 0.01 and *p* < 0.05, respectively).

Given that a high sugar intake promotes oxidative stress in cells, changes in the activity of enzymes crucial for antioxidative defence were examined (Figure 6E). Superoxide dismutase (SOD) serves as the first line of defence, converting superoxide radicals into oxygen and hydrogen peroxide. A significant main effect of the high-dextrose drinking regimen on reduced total SOD activity was observed (*p* < 0.05), and a significant main effect of maturity on increased activity of its mitochondrial isoform, manganese SOD (MnSOD), was found (*p* < 0.01) in the middle-aged adult groups. In contrast to total SOD and MnSOD, copper-zinc SOD (CuZnSOD) activity was independently affected by maturity (*p* < 0.001), dextrose regimen (*p* < 0.001), and by a significant interaction between maturity and dextrose regimen (*p* < 0.001). The main effect of dextrose regimen was observed as decreased catalase (CAT) activity (*p* < 0.05), with the lowest descriptive CAT activity recorded in the 60D middle-aged adult group. Additionally, a significant decrease in CAT activity was found in this group compared with its corresponding untreated control (*p* < 0.05).

Besides SOD, the maintenance of cellular redox balance depends on enzymes that use glutathione (GSH) to neutralise free radicals, peroxides, and heavy metals. Accordingly, the activities of glutathione S-transferase (GST), glutathione peroxidase (GPX), and glutathione reductase (GR) were investigated. Two-way ANOVA revealed a significant main effect of maturity (*p* < 0.01) and a significant main effect of dextrose regimen (*p* < 0.01) on decreased GST activity. GST catalyses the conjugation of GSH to reactive oxygen species and is one of the major 4-HNE-metabolising pathways. In addition, a main effect of maturity (*p* < 0.05) was found on GR activity, which is responsible for GSH recycling, with the same 60D middle-aged adult group also exhibiting the lowest descriptive GST activity. Finally, two-way ANOVA showed a significant main effect of dextrose regimen on GPX activity (*p* < 0.05). These patterns suggest that a long-term high-dextrose drinking regimen coincides with a weakened antioxidant profile, particularly within the middle-aged adult rats (Figure 6E).

### 2.7. Dose- and Age-Dependent Effects of a High-Dextrose Drinking Regimen on the Subcellular Distribution and Expression of HMGB1, NF-κB p65, and Nrf2

**Figure 7:**
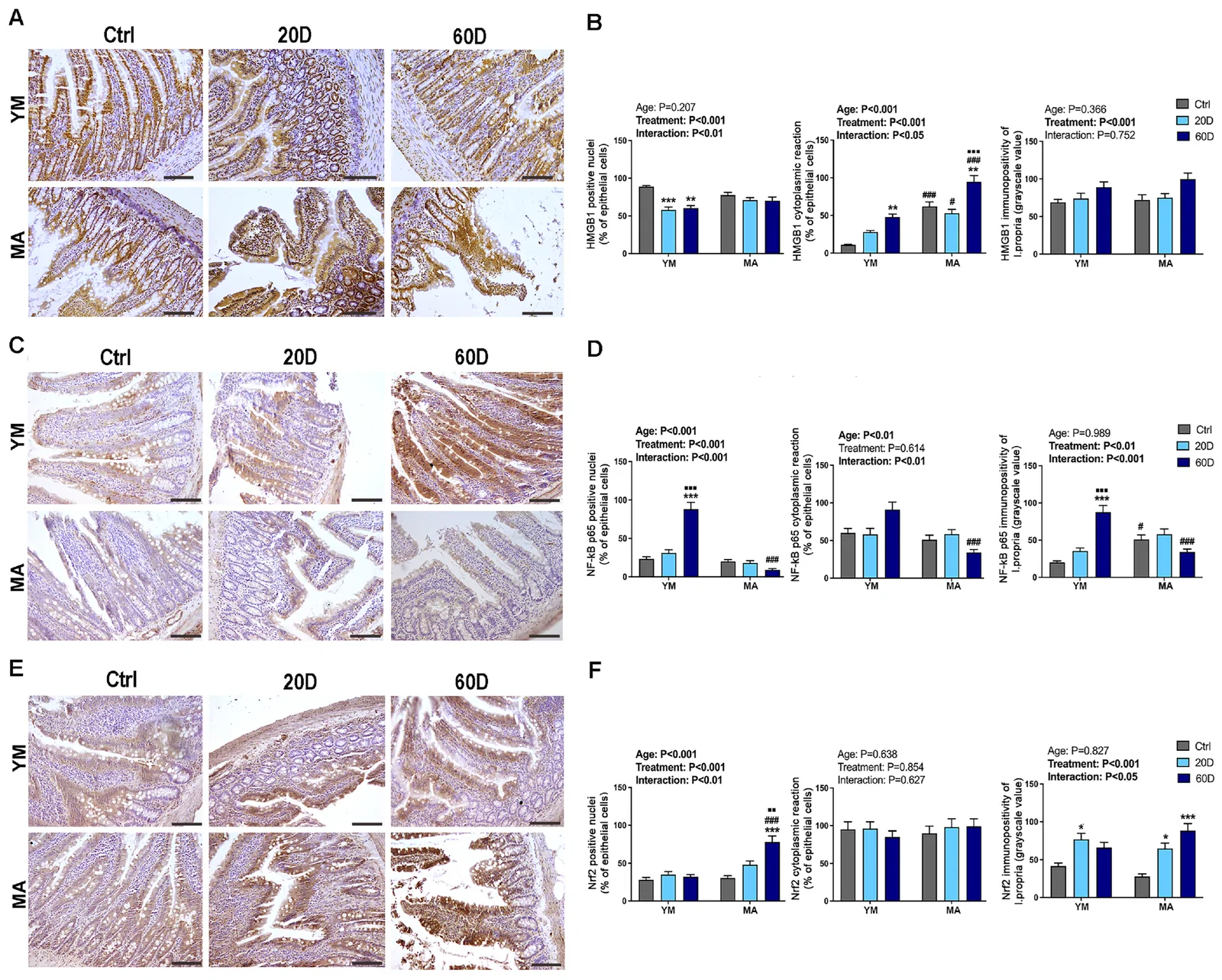
Subcellular distribution and expression of high-mobility group box 1 (HMGB1) protein, nuclear factor-κappa B (NF-κB) p65, and nuclear factor erythroid 2-related factor 2 (Nrf2) in the small intestine of young-maturing (YM) and middle-aged (MA) adult rats following a high-dextrose drinking regimen. (**A**,**C**,**E**) Representative immunohistochemical micrographs showing tissue localization of HMGB1, NF-κB p65, and Nrf2 under control (Ctrl), 20% dextrose (20D), and 60% dextrose (60D) treatment (×20, scale bar: 100 µm). (**B**,**D**,**F**) Bar graphs quantifying nuclear positivity (%), cytoplasmic reaction (%), and lamina propria grayscale intensity for each protein. Statistical analyses were performed using an independent-measures two-way ANOVA followed by Tukey’s post hoc test, with the resulting *p*-values for maturity, dextrose treatment, and interaction factors indicated above each graph. Values are expressed as mean ± SEM (n = 3 independent biological replicates per group, with each value derived from 10 non-overlapping technical microscopic fields per animal). Comparison of treated groups with the corresponding control group (*); comparison of rats at different stages of maturity under the same dextrose drinking conditions (#); comparison of rats at the same maturity stage under different dextrose drinking regimens (▪). Statistical significance was set at *p* < 0.05, with *, #, *p* < 0.05; **, ▪▪ *p* < 0.01; ***, ###, ▪▪▪ *p* < 0.001.

HMGB1, NF-κB p65, and Nrf2 are involved in the cellular response to stress and often act together to manage damage caused by stressors such as inflammation. As high sugar intake causes rapid changes in blood glucose and may stimulate the tissue immune response, creating conditions in which inflammatory strain can persist, these three proteins were examined using immunohistochemistry. Under stress conditions, HMGB1 translocates from the nucleus to the cytoplasm and is eventually secreted as a signal of cell injury or necrotic death. Microscopic analysis showed that HMGB1 (Figure 7A) is mainly localised within the nuclei of epithelial cells in both control groups. The high-dextrose drinking regimen resulted in increased immunopositivity in the cytoplasm of epithelial cells compared with the appropriate controls, accompanied by a decrease in nuclear HMGB1 positivity. In addition, a significant main effect of dextrose regimen (*p* < 0.001) was detected on immunopositivity within the lamina propria.

Statistical analysis (Figure 7B) showed a significant main effect of the dextrose regimen on the reduction in HMGB1-positive nuclei (*p* < 0.001) and a significant interaction between maturity and dextrose regimen (*p* < 0.01). Tukey’s post hoc analysis revealed that 20D and 60D young maturing rats had a lower percentage of HMGB1-immunopositive nuclei than their corresponding untreated control group (*p* < 0.001 and *p* < 0.01, respectively). Furthermore, there was a significant main effect of maturity on the percentage of epithelial cells exhibiting a positive cytoplasmic reaction (*p* < 0.001), a significant main effect of dextrose regimen (*p* < 0.001), and a significant interaction between maturity and dextrose regimen (*p* < 0.05). The post hoc test revealed a statistically significant increase in the percentage of epithelial cells with a positive cytoplasmic reaction in the 60D young maturing rats and in the middle-aged control group, respectively, compared with the young control rats (*p* < 0.01 and *p* < 0.001, respectively). A significant increase was also observed in the 60D middle-aged adult rats compared with their corresponding untreated control (*p* < 0.01) and the 20D middle-aged group (*p* < 0.001). The highest percentage of epithelial cells with HMGB1-immunopositive cytoplasm was recorded in the 60D middle-aged adult group, and Tukey’s post hoc analysis revealed a statistically significant difference when compared with their young counterparts (*p* < 0.001).

Activation and translocation of NF-κB p65, a key transcription factor regulating genes for pro-inflammatory cytokines, to the nucleus indicates that the cell is responding to inflammatory or oxidative stress. Immunohistochemical analysis of NF-κB p65 (Figure 7C) showed that the highest immunoexpression of NF-κB p65 was present in the nuclei, as well as in the cytoplasm and lamina propria, in 60D young maturing rats. These results suggest that NF-κB p65 signalling is prominently activated, with elevated tissue expression levels following excessive dextrose consumption in young maturing rats. Quantitative analysis and two-way ANOVA supported these microscopic observations (Figure 7D). There was a significant independent main effect of maturity (*p* < 0.001) on NF-κB p65 nuclear immunoexpression, a significant independent effect of dextrose regimen (*p* < 0.001), and a significant interaction between maturity and dextrose regimen (*p* < 0.001). Tukey’s post-hoc test showed a significant increase in nuclear NF-κB p65 immunoexpression in the 60D young maturing rats compared with their corresponding control and the 20D young maturing rats (*p* < 0.001 for both). A statistically significant decrease was observed in the 60D middle-aged adult group compared with the 60D young maturing rats (*p* < 0.001). Furthermore, two-way ANOVA showed a significant independent main effect of maturity (*p* < 0.01) on the percentage of cells with NF-κB p65 cytoplasmic immunoexpression, alongside a significant interaction between maturity and dextrose regimen (*p* < 0.01). A significant decrease in the percentage of epithelial cells with a positive cytoplasmic reaction was noted in the 60D middle-aged adult rats compared with their young counterparts (*p* < 0.001). Finally, there was a significant independent main effect of dextrose regimen (*p* < 0.01) on NF-κB p65 immunoexpression within the lamina propria, as well as a significant interaction between maturity and the drinking regimen (*p* < 0.001). The post-hoc test showed a significant increase in the 60D young maturing rats compared with their corresponding control (*p* < 0.001) and compared with 20D young maturing rats (*p* < 0.001), in the middle-aged control rats compared with their younger counterparts (*p* < 0.05), and in the 60D middle-aged adult rats compared with the 60D young rats (*p* < 0.01).

While HMGB1 and NF-κB p65 drive inflammation, Nrf2 serves as a primary antioxidant marker and regulator of redox homeostasis. Its activation and nuclear localisation indicate that the cell is likely attempting to defend itself against oxidative stress and associated inflammation. Nrf2 cytoplasmic and nuclear immunopositivity was observed across all groups, with a particularly notable nuclear reaction documented in the 60D middle-aged adult rats (Figure 7E). Two-way ANOVA showed a significant independent main effect of maturity (*p* < 0.001) on Nrf2 nuclear immunoexpression, a significant independent main effect of dextrose regimen (*p* < 0.001), and a significant interaction effect between maturity and dextrose regimen (*p* < 0.01) (Figure 7F). Post hoc testing showed a significant increase in the percentage of Nrf2-immunopositive nuclei within the 60D middle-aged adult rats compared to their corresponding untreated control group, the young controls (*p* < 0.001 for both), and the 20D middle-aged adult rats (*p* < 0.01). For the percentage of epithelial cells with an Nrf2-positive cytoplasmic reaction, two-way ANOVA revealed no significant main or interaction effects. Furthermore, two-way ANOVA showed a significant independent main effect of dextrose regimen (*p* < 0.001) on Nrf2 immunoexpression within the lamina propria, alongside a significant interaction effect between maturity and dextrose regimen (*p* < 0.05). Tukey’s post hoc test demonstrated increased Nrf2 immunoexpression in the lamina propria of both the young maturing and middle-aged adult rats exposed to the 20% dextrose drinking regimen compared to their respective untreated controls (*p* < 0.05). There was also a significant increase in Nrf2 immunoexpression within the lamina propria of the 60D middle-aged adult rats compared to their age-matched control (*p* < 0.001).

## 3. Discussion

In this study, we demonstrated that an 8-week high-dextrose drinking regimen causes dose- and maturity-dependent structural and functional changes in the small intestine. Specifically, middle-aged adult rats exhibited the most pronounced alterations, characterized by mucosal barrier disruption, tissue iron accumulation, and altered transcription factor profiles, despite maintaining stable fasting HOMA-IR values. This dissociation between basal systemic insulin sensitivity and localized small-intestinal homeostasis highlights how dextrose overexposure drives tissue-specific responsiveness across distinct stages of biological maturity.

Both iso-caloric and hyper-caloric high-carbohydrate diets primarily increase substrate availability, as excessive simple sugar consumption upregulates multiple metabolic pathways. In experimental hyper-caloric models, sugar (mainly fructose or sucrose) is typically added to drinking water. This reduces solid food intake but increases total caloric consumption compared with animals fed standard chow [27,28]. In the present study, eight weeks of exposure to a high-dextrose drinking regimen did not alter basal insulin resistance, as indicated by stable HOMA-IR values in both young adult and middle-aged adult rats. This stability indicates a robust systemic adaptive capacity. However, this homeostatic preservation is age-dependent. Younger rats adapt by reducing food intake, which leads to a slight decrease in body weight. In contrast, middle-aged rats likely partition excess energy into adipose tissue, resulting in a linear increase in body mass and signalling a long-term risk of metabolic disorders. This fat storage may function as a compensatory defence mechanism that prevents toxic elevations of free glucose, thereby delaying the onset of insulin resistance.

While systemic fasting HOMA-IR values remained stable, they do not necessarily indicate an absence of localised tissue stress. Although the high-dextrose drinking regimen had no effect on fasting blood glucose, insulin levels, or HOMA-IR, the IP-GTT results revealed early impairment of glucose handling in middle-aged rats exposed to high dextrose. This impairment became statistically evident when their AUC values were compared with those of younger rats rather than age-matched controls, confirming that middle-aged rats have a compromised capacity to manage high dextrose intake, consistent with the literature showing that glucose intolerance increases with age [29,30,31]. Structurally, fasting HOMA-IR measures basal sensitivity, while the IP-GTT dynamically assesses acute sugar clearance. This post-challenge glucose intolerance is a well-documented physiological precursor of systemic insulin resistance. [32]. When interpreting these small-intestinal changes, we must consider that a concentrated dextrose drinking regimen imposes immediate, direct challenges on the epithelium, including high osmotic pressure and saturation of hexose transporters. Concurrently, because the IP-GTT relies on systemic administration, these results reflect alterations in peripheral glucose handling rather than direct changes in luminal transport or mucosal hormone kinetics. At the same time, systemic metabolic changes induced by chronic dextrose overexposure, such as postprandial glucose intolerance, can further exacerbate this tissue stress, potentially accelerating mucosal alterations as biological maturity advances [33].

Although high-sugar or high-fat diets are known to alter gut morphology, microbiota and permeability [7,34,35], our findings demonstrate that small intestinal morphometric parameters are predominantly driven by age. Specifically, villus height, crypt depth and muscle layer thickness varied with age, whereas villus surface area (VSA), an indicator of absorptive capacity, showed a distinct age–treatment interaction and reached the highest value in young maturing, untreated rats. Although the literature indicates that intestinal surface area expands during early life before stabilising or declining with age [35,36,37,38,39], our study revealed a novel, maturity-dependent response to dextrose. Middle-aged adult rats on the dextrose regimen tended to increase their absorptive surface area, whereas young maturing rats exhibited the opposite trend. This divergence likely reflects age-related changes in glucose transport capacity, which typically declines with maturity [40]. Consequently, young maturing rats may downregulate their surface area to compensate for higher baseline glucose absorption rates, whereas adults may expand it to offset reduced transport efficiency. These findings underscore that structural adaptations to dextrose overconsumption are strongly age-dependent, so data from young models cannot be directly extrapolated to adults.

Beyond morphometric changes, we observed distinct alterations in epithelial cell composition. In adult rats on the high-dextrose regimen, the proportion of goblet cells (GCs) decreased, whereas intraepithelial lymphocytes (IELs) increased. GCs are essential for mucosal immunity, secreting mucins, chemokines and cytokines that constitute the first line of defence [40]. Thus, their reduction may compromise barrier integrity, increasing susceptibility to injury and inflammation. Although Alcian blue staining provides a static structural overview rather than capturing dynamic mucin secretion rates or mucus layer thickness, the reduced GC counts in the 60D middle-aged adult group strongly indicate a fundamental shift in mucosal secretory capacity and a compromised physical barrier. Concurrently, an elevated proportion of IELs above the epithelial basement membrane was detected in both young maturing rats on the high-dextrose regimen and middle-aged adults across both dextrose doses, indicating activation of local immune responses. This is consistent with findings by Min et al. [41], who reported elevated serum IL-6 and TNF-α levels alongside intestinal mucosal damage following a two-week dietary glucose infusion in rats. Ultimately, high-carbohydrate diets rich in refined sugars are well-known triggers of pro-inflammatory pathways such as NF-κB p65, which stimulate ROS production and drive chronic, low-grade intestinal inflammation [1].

Our findings indicate that dextrose overconsumption alters the biochemical and structural properties of the small intestine in an age- and dose-dependent manner. The most pronounced perturbations occurred in middle-aged adult rats on the 60% dextrose regimen. Typically, the small intestine rapidly adapts to dietary challenge via tightly regulated epithelial renewal, in which high crypt cell proliferation is balanced by programmed cell death. In our study, exposure to the high-dextrose regimen induced a dose-dependent reduction in Ki-67-positive nuclei across both investigated age groups, indicating suppressed proliferative activity independent of animal maturity. This aligns with the established timeline of epithelial turnover, which shifts from rapid developmental expansion in youth to steady-state maintenance in adulthood, and may further decline with age [42,43,44]. Concurrently, middle-aged rats on the 60% dextrose regimen exhibited a significant increase in the rate of cell death, characterised by apoptotic and necrotic morphology, indicative of severely compromised intestinal turnover. In young maturing rats, this combination of diminished proliferation and an elevated cell death is likely to drive the observed villous atrophy, crypt shortening, and reduced absorptive surface area. Conversely, dextrose intake in middle-aged adult rats prompts structural remodelling that expands the VSA, a feature that could facilitate systemic dextrose influx and a subsequent increase in body weight. Crucially, at the 60% dextrose regimen, this remodelling becomes pathological, triggering extensive epithelial apoptosis, necrosis, and a profound loss of protective GCs. This tissue-level deterioration shows that stable systemic markers, such as HOMA-IR indices, can mask local tissue stress.

It is widely accepted that dietary components modulate intestinal barrier integrity and alter intestinal physiology. This barrier provides robust protection through various mechanisms, including the mucus layer, regenerative capacity, and tight junctions. While intestinal barrier function is regulated by a complex network of multiple interacting proteins, including occludin, claudins, and ZO-1, we focused on JAM-1 because of its critical role in regulating epithelial permeability, inflammation, and proliferation, which are central to our study. JAM-1 is a highly responsive transmembrane protein essential for intestinal wound repair in vivo, serving as an early indicator of epithelial strain, leukocyte infiltration, and the initial phases of barrier breakdown [45]. In this study, both rat maturity and a high-dextrose drinking regimen increased JAM-1 protein expression. However, the localised morphological changes observed via H&E staining, together with increased static JAM-1 expression, indicate a structurally compromised barrier rather than a direct functional increase in paracellular permeability. Specifically, the elevated JAM-1 in the 60D middle-aged rats may also suggest localised compensatory remodelling or shifts in epithelial cell composition under chronic carbohydrate saturation. While tight junction disruption and altered enterocyte morphology typically lead to functional impairment [28,41,46], the absence of dynamic in vivo permeability assays, such as FITC–dextran gavage or circulating markers of bacterial translocation, remains a limitation of this study. Additionally, evaluating other components of the tight junction network is necessary to fully characterise barrier degradation. Nonetheless, the concurrent reduction in GCs density, localised cellular damage, and pronounced infiltration of IELs observed in our study collectively indicate structural damage.

The observed increase in JAM-1 expression was unexpected, as high-carbohydrate regimens and hyperglycaemia are typically associated with downregulation of tight junction proteins, leading to increased paracellular permeability [46,47]. Although this elevated expression may suggest an active enterocyte compensatory mechanism preserving barrier integrity, methodological limitations must be considered. Because our Western blot analyses quantified the total protein pool of intestinal homogenates, the elevated JAM-1 signal could partly originate from apoptotic epithelial cells or infiltrating immune cell populations. Under metabolic strain, microscopic gaps can form within the epithelial barrier due to accelerated enterocyte apoptosis and necrosis. The remaining enterocytes may transiently upregulate JAM-1 to seal these spaces and maintain mucosal structural integrity. In this context, the linear increase in JAM-1 expression following dextrose overexposure in the middle-aged group likely represents a compensatory defence mechanism activated in response to enterocyte loss and concurrent degradation of the protective mucus layer. Furthermore, JAM-1 is highly expressed in endothelial cells and leukocytes, where it regulates cellular migration and inflammatory signalling [48,49]. Therefore, the increased expression may partly reflect immune cell infiltration, which is consistent with the elevated numbers of IELs observed in our dextrose-treated groups. Future immunofluorescence studies tracking localised, membrane-specific JAM-1 distribution at intercellular contact zones will be valuable for clarifying this mechanism.

Beyond JAM-1, other markers of intestinal function were altered by rat maturity and dextrose drinking regimen. Immunohistochemistry revealed increased serotonin and NSE expression in middle-aged rats on the 60D regimen. Enteric serotonin regulates gut sensory-motor, secretory, and metabolic functions [50,51]. Given the structural alterations observed here, namely mucosal damage, accelerated enterocyte apoptosis and necrosis, and intraepithelial lymphocyte infiltration in the middle-aged group, elevated serotonin expression likely serves as a key neuroendocrine modulator [52,53,54]. Specifically, increased serotonin signalling can stimulate enteric neurons, altering motility and tissue secretion, while simultaneously interacting with resident receptors to drive local immune regulation and sustain chronic inflammation in the small intestine. Parallel to the serotonin increase, the dextrose-related rise in NSE expression further indicates neuroendocrine activation. Although this elevated expression primarily reflects hypersecretion by EECs and enteric neurons, the greater presence of necrotic cells suggests that passive leakage from disrupted membranes also contributes to these tissue levels [55]. Therefore, increased serotonin and NSE levels in response to a chronic high-dextrose drinking regimen likely result from a combination of active neuroendocrine responses and passive cellular release driven by structural tissue damage [55,56]. However, NSE is a cytosolic glycolytic enzyme and a recognised marker of oxidative stress-induced neurodegeneration, so it is not exclusively specific to enteric neurons [56]. It can also be expressed by non-neuronal populations, such as smooth muscle cells or infiltrating leukocytes under metabolic strain, which restricts its utility as a definitive marker of enteric nervous system remodelling. This relationship between local NSE tissue expression and glucose-handling strain warrants further investigation. Ultimately, it remains to be determined whether this elevated NSE expression serves a compensatory protective role, indicates enteric neuronal injury, or reflects both mechanisms simultaneously.

Intestinal glucose absorption is essential for postprandial homeostasis and depends heavily on mucosal exposure in the upper gastrointestinal tract [57]. Apical translocation of the transporter GLUT2 is a well-known adaptive mechanism triggered by high luminal glucose [58]. Consistent with this, the high-dextrose drinking regimen significantly altered intestinal GLUT2 expression, promoting its apical localisation, most notably in 60D young maturing rats. Interestingly, we also detected GLUT2 expression within lamina propria leukocytes following the high-dextrose regimen, a phenomenon not previously reported. To investigate the energetic cost of these changes, we evaluated ATP synthase expression and localisation. A distinct age–treatment interaction revealed elevated ATP synthase expression in the same lamina propria regions harbouring GLUT2-positive leukocytes. This concurrent upregulation suggests heightened metabolic and energetic demands in infiltrating immune cells, aligning with reports that inflammatory conditions accelerate T-lymphocyte activation and glycolytic flux [59,60]. Mechanistically, intracellular glucose accumulation increases ATP production, which can paradoxically elevate ROS and oxidative stress. Under hypermetabolic conditions, excess ATP is released into the extracellular space, where it acts as a DAMP [61]. This extracellular ATP activates the P2X7 receptor on immune cells, driving their activation and initiating a pro-inflammatory cascade [62,63]. Taken together, these findings indicate that chronic dextrose overconsumption alters mucosal ATP dynamics, directly linking metabolic overload to intestinal immune activation and inflammatory responses.

In addition to extracellular ATP, local immune activation can be driven by HMGB1, another prominent DAMP molecule. In this study, both rat maturity and dextrose treatment promoted nuclear-to-cytoplasmic HMGB1 translocation, an effect most pronounced in 60D middle-aged rats. Notably, this group also exhibited the strongest HMGB1 immunopositivity within the lamina propria, a localization poised to amplify innate immune responses and fuel intestinal inflammation via the TLR4/NF-κB p65 pathways. These expression patterns strongly implicate HMGB1 in glucose intolerance, aligning with documented elevations of serum HMGB1 in diabetic and insulin-resistant models [64]. Beyond its pro-inflammatory role, extracellular HMGB1 can directly impair insulin signaling through TLR4 and RAGE pathways, potentially compounding the metabolic strain observed in glucose handling [65,66]. Given that extracellular HMGB1 release is a marker of necrotic cell death [67], these molecular changes closely mirror our morphological observations of substantial enterocyte necrosis in the 60D middle-aged group. The recent literature indicates that cytoplasmic and extracellular HMGB1 can modulate and amplify ferroptotic signalling, a regulated, iron-dependent form of cell death driven by depleted antioxidant defences and accelerated membrane lipid peroxidation [20,68]. Our findings align with these iron-dependent pathways, as evidenced by localised tissue iron accumulation, a significant increase in the lipid peroxidation marker 4-HNE, and compromised antioxidant capacity. This increased tissue iron retention potentially catalyses the Fenton reaction, generating excessive ROS, driving lipid peroxidation, and thereby raising the possibility of triggering a ferroptotic cascade. Taken together, these patterns suggest that nucleocytoplasmic shifting of HMGB1 does not merely reflect general cellular disturbance but instead correlates with a maturity-dependent coordination of concurrent events, including distinct modes of cell death, inflammatory pathways, and antioxidant responses within the small intestine.

The transcription factor NF-κB p65 drives intestinal inflammation by orchestrating mucosal immune responses upon nuclear translocation [69,70,71]. Our study demonstrates an age-dependent NF-κB p65 response to dextrose overconsumption, particularly under the 60D regimen. Specifically, nuclear NF-κB p65 expression increased in young maturing rats but declined in middle-aged animals. This downregulation in the middle-aged group coincided with elevated apoptotic cell death, illustrating the context-dependent role of NF-κB p65 in cell fate. Mechanistically, transcriptionally active NF-κB p65 maintains epithelial survival by upregulating anti-apoptotic proteins such as Bcl-2 [72]. Accordingly, our findings revealed reduced Bcl-2 expression under the 60D regimen in middle-aged rats. This nuclear loss of NF-κB p65 compromises the anti-apoptotic pathway, increasing epithelial vulnerability to caspase activation and apoptosis. Concurrently, reduced NF-κB p65 immunoexpression in the lamina propria suggests a localised dampening of inflammatory signalling within this immune compartment. Interestingly, while the 60% dextrose regimen depleted nuclear NF-κB p65 levels in middle-aged adult rats, it simultaneously elevated nuclear Nrf2 levels, which is the opposite of the expression profile observed in younger animals. Because NF-κB p65 and Nrf2 functionally cross-regulate and compete for the same transcriptional co-factors, the p65 subunit typically acts to suppress Nrf2 transcription. [69,73]. Thus, our results suggest that the reduced nuclear NF-κB p65/Nrf2 ratio observed in the 60D middle-aged group may reflect a state of impaired inflammatory responsiveness rather than a successful adaptive defence. Together with the concurrent accumulation of 4-HNE and tissue iron, this altered signalling profile likely indicates a state of signalling weakness in the small intestine under conditions of chronic dextrose overexposure. Although we observed simultaneous changes in the subcellular distribution and activation profiles of HMGB1, NF-κB p65, and Nrf2, these descriptive data do not establish direct causal interactions or precise feedback mechanisms among them. Instead, these concurrent alterations likely reflect a broad cellular response to chronic dextrose overexposure. Additional mechanistic studies are therefore needed to clarify the regulatory networks governing these redox-sensitive proteins during dextrose-induced small-intestinal strain. Additionally, the lack of direct gut microbiota analysis remains another limitation of this study. Although gut microbiota sequencing was not performed in this study, high-sugar diets are well-documented to induce rapid dysbiosis [74,75]. For example, the depletion of symbiotic taxa reduces protective short-chain fatty acids, while expansion of pathobionts can promote epithelial ROS, impair GCs, and alter downstream inflammatory pathways. Consequently, validating these microbial contributions through comprehensive sequencing remains for future studies.

Given the many functions of the intestinal epithelium, it is continually exposed to free radicals produced during oxidative reactions, especially during food intake, digestion, and absorption. To preserve intestinal homeostasis, it expresses a range of antioxidant enzymes [76]. In our study, the dextrose drinking regimen reduced total small intestinal SOD and CuZnSOD activities while slightly increasing MnSOD in middle-aged rats compared with younger counterparts. These divergent changes likely reflect distinct cytoplasmic and mitochondrial responses, as high dextrose could induce superoxide overproduction and reduce CuZnSOD activity. The decline in total SOD activity may stem from glycation and subsequent inactivation of CuZnSOD, directly compromising antioxidant complexes [77,78]. Consequently, hydroxyl radicals produced from glycated CuZnSOD, combined with depleted CAT activity, likely drive small intestinal changes in middle-aged rats on the high-dextrose drinking regimen [78,79,80]. Interestingly, dextrose treatment upregulated GPx, particularly in middle-aged rats, indicating an enhanced adaptive capacity to neutralise H_2_O_2_. To inactivate H_2_O_2_, GPx and GR function as a coupled antioxidant system, since GPx uses reduced GSH to catalyse the reduction of H_2_O_2_ to water, while GR regenerates glutathione (GSH) from its oxidised form to maintain the GSH level [81,82]. In contrast to GPx, dextrose treatment reduced GR activity due to both age and dextrose. This divergent response suggests that while the upregulation of GPx reflects an adaptive attempt to neutralise H_2_O_2_, the concurrent decline in GR activity renders this defence unsustainable. Consequently, the breakdown of this coupled system likely leads to critical depletion of intracellular GSH levels. This metabolic exhaustion not only compromises the tissue’s capacity to handle sustained oxidative stress but also enhances small intestinal susceptibility to lipid peroxidation and probably to downstream ferroptotic signalling in older, middle-aged rats. Following a similar pattern, GST activity, which is responsible for metabolising 4-HNE, was also reduced by both age and dextrose [79,83]. This reduction closely aligns with the elevated 4-HNE levels observed in middle-aged rats, emphasising the loss of GST-mediated protection against lipid peroxidation.

## 4. Materials and Methods

### 4.1. Animals and Experimental Procedure

Male Wistar rats were bred and housed in the animal facility of the Institute for Biological Research “Siniša Stanković”, National Institute of the Republic of Serbia (IBISS), Belgrade, Serbia. Commercial food (Veterinarski Zavod Subotica, Victoria Group, Subotica, Serbia) and drinking water were available ad libitum. The animals were kept at a controlled ambient temperature (22 ± 2 °C) under a standard 12-h light/dark cycle. All animal procedures complied with Directive 2010/63/EU on the protection of animals used for experimental and other scientific purposes and were approved by the Ethics Committee for the Use of Laboratory Animals of the Ministry of Agriculture, Forestry and Water Management of the Republic of Serbia (reference number: 323-07-04571/2020-05). At the start of the 8-week experiment, rats at 1 month of age (young, maturing) and 14 months of age (older, middle-aged) were randomly divided into three subgroups (n = 9 per subgroup): the untreated control group (Ctrl), maintained on standard laboratory rat chow and tap water; the 20% dextrose-treated group (20D), which received the same chow and a 20% dextrose solution instead of drinking water; and the 60% dextrose-treated group (60D), which received the same chow alongside a two-bottle system containing the 60% dextrose solution and plain tap water ad libitum. The two-bottle system provided animals with continuous access to plain water, preventing systemic dehydration. At the same time, voluntary water intake likely diluted the intestinal contents, thereby reducing the luminal hyperosmotic stress associated with a high-dextrose drinking regimen. The theoretical osmolality of the administered drinking solutions was approximately 1110 mOsm/kg H_2_O for the 20D solution and exceeded 3300 mOsm/kg H_2_O for the 60D solution. At the time of sacrifice and tissue analysis, the young, maturing rats had reached 3 months of age (young adults), while the older rats had reached 16 months of age (middle-aged adults). The rats were housed 2–3 per cage, and daily food and liquid intake per cage were measured during the eight weeks of the experiment. Body mass was measured once per week. Total energy intake was expressed as kJ per day per animal. Daily energy intake for animals on the standard diet was calculated as food weight (g) per day per animal × 14.05 kJ, while energy intake for rats on the high dextrose-drink regimen was calculated as the sum of the calories ingested as food and dextrose solution (food weight (g) per day per animal × 14.05 kJ + dextrose intake (mL) per animal × 2.85 kJ or 8.54 kJ for 20% and 60% dextrose, respectively). Actual dextrose intake was further normalized to body mass and expressed as g/kg/day, using the mean body mass calculated across all weekly measurements, and the percentage of total daily caloric intake derived from the dextrose solutions was calculated for each experimental unit (cage). Detailed overview of experimental units, endpoind category and statistical model is presented in Supplementary Table S1.

### 4.2. Intraperitoneal Glucose Tolerance Test (IP-GTT) and Biochemical Analysis

To evaluate systemic peripheral glucose clearance capacity independently of confounding gastrointestinal factors such as gastric emptying rates, mucosal transporter kinetics, and the gut–incretin axis, which are typically assessed by an oral glucose tolerance test (OGTT), the intraperitoneal glucose tolerance test (IP-GTT) was selected for this study. IP-GTT was performed during the final week of the experiment. Rats were fasted overnight (with water available ad libitum, excluding dextrose solutions) and received an intraperitoneal injection of glucose (2 g/kg). To determine glucose concentration, blood samples were collected from the tip of the tail immediately before the glucose injection (time 0) and at 15, 30, 60, 90, and 120 minutes after injection. Blood glucose levels were measured using a glucometer and AccuChek Active strips (F. Hoffmann-La Roche AG, Mannheim, Germany). The AUC was calculated using the trapezoidal rule [84]. At the end of the experiment, animals were rapidly decapitated with a guillotine (Harvard-Apparatus, Holliston, MA, USA), and blood and small intestine (proximal jejunum) samples were collected and routinely processed for biochemical, microscopic, immunoblot, and spectrophotometric analyses. After blood collection, serum was prepared and stored at −80 °C for further analysis. Serum insulin levels were measured by radioimmunoassay (INEP, Belgrade, Serbia). Using fasting plasma glucose and fasting plasma insulin concentrations, HOMA-IR, a marker of insulin sensitivity, was calculated according to the following formula: HOMA-IR = [FPG (mg/dL) × FPI (μU/mL)]/2430, where 2430 is a constant derived from the assumption that the HOMA-IR of young and adult rats averages 1 [85].

### 4.3. Histological, Histochemical and Morphometric Analysis

Immediately after dissection, pieces of proximal jejunum were fixed overnight in 4% formaldehyde at 4 °C and routinely processed for embedding in paraffin blocks, as previously described. Well-oriented, longitudinally cut 5-µm-thick tissue sections were deparaffinised and rehydrated before staining. For routine histological and morphometric analysis of the small intestine, slides were stained with haematoxylin and eosin (H&E) [86]. Additionally, Alcian blue/nuclear fast red staining was performed to visualise mucin in GCs. To detect iron (Fe^3+^) accumulation, DAB (3,3′-diaminobenzidine)-enhanced Perl’s Prussian blue staining was performed [80], with a positive reaction detected as brown intracellular accumulation. The staining intensity reflecting iron accumulation was scored as follows: 0—absent, 1—mild, 2—moderate, 3—massive. Samples were mounted in dibutyl phthalate polystyrene xylene (DPX) medium (Sigma-Aldrich, St. Louis, MO, USA) and examined with a DMLB microscope (Leica Microsystems, Wetzlar, Germany) [86]. Morphometric analysis was performed to establish the following parameters: villus height, crypt depth, villus width (at mid-villus height), and muscle layer thickness. The results obtained by measuring villus height and crypt depth were used to calculate VSA using the equation: VSA = (2π) × (Vw/2) × (Vh), where π = 3.14, Vw = villus width at mid-villus height, and Vh = villus height [87]. To determine the proportion of enterocytes, GCs, and IELs in intestinal villi, a counting method was used. For all these analyses, 30 randomly selected micrographs per group (10 per animal, three animals per group) were used.

### 4.4. Immunohistochemical (IHC) and Immunofluorescence Analysis

To detect the expression and localisation of proteins of interest (Ki-67, serotonin, NSE, HMGB1, NF-κB p65, and Nrf2), as well as the formation of 4-HNE in the small intestine, immunohistochemistry was performed. Sections were deparaffinised with xylene and rehydrated in graded ethanol. Antigen retrieval, blocking of endogenous peroxidase activity, and blocking of non-specific binding with bovine serum albumin were performed as described previously [86] before incubation with primary antibodies. Samples were then incubated overnight at 4 °C with the following primary antibodies: anti-Ki-67 (ab16667, 1:500), anti-HMGB1 (ab18256, 1:1000), anti-NF-κB p65 (ab7970, 1:200), anti-Nrf2 (ab31163, 1:200), anti-4-HNE (ab46545, 1:500), all purchased from Abcam (Cambridge, UK), and anti-serotonin (M0758, 1:100) and anti-NSE (M0873, 1:100) purchased from DAKO (Carpinteria, CA, USA). After rinsing with PBS, the sections were incubated with appropriate secondary antibodies, goat anti-rabbit (ab97051, 1:1000, Abcam) and horse anti-mouse (7076S, 1:1000, Cell Signaling Technology, Danvers, CO, USA), for 1 h at room temperature. The final reaction product was visualised with DAB chromogen solution (K 3408, Dako liquid DAB+ Substrate Chromogen System, Carpinteria, CA, USA). After counterstaining with haematoxylin, slides were mounted in DPX and examined with a DMLB microscope (Leica Microsystems, Wetzlar, Germany). To quantify IHC staining, approximately 30 micrographs (10 per animal, three animals per group) were analysed. Two metrics for IHC staining were used: the percentage of positive nuclei/cells (counting method) and staining intensity. For quantification of nuclear staining, the total number of nuclei was used to calculate the percentage of epithelial cells in villi exhibiting nuclear staining. For quantification of staining immunopositivity, micrographs were analysed in ImageJ software (ImageJ version 1.53e, National Institutes of Health, Bethesda, MA, USA) using the Colour Deconvolution2 plugin, in which images were split into haematoxylin and DAB channels [88]. Obtained greyscale values (from 0 = black—the strongest signal, to 255 = white—no signal) were subtracted from 255 to obtain values directly proportional to immunopositivity strength.

Immunofluorescence labelling was used to examine the localisation of GLUT2 and ATP synthase, as well as their protein expression in the lamina propria, using anti-GLUT2 (ab192599, 1:100) and anti-ATPB (ab14730, 1:500), purchased from Abcam (Cambridge, UK). After routine deparaffinisation, rehydration, antigen retrieval with citrate buffer, and protein blocking (5% BSA in PBS), primary antibodies were applied and incubated overnight at 4 °C. After thorough washing, the sections were incubated with the appropriate secondary antibodies: anti-mouse Alexa Fluor 488 (1:200; ab150113, Abcam) and anti-rabbit Alexa Fluor 647 (1:200; ab150079, Abcam). Antibodies were diluted in 1% BSA in PBS and incubated for 60 min at room temperature. Nuclei were stained with Sytox Orange (1:1000 in PBS, Thermo Fisher Scientific, Carlsbad, CA, USA). After washing, sections were embedded in Mowiol (Sigma-Aldrich, St. Louis, MO, USA) and analysed on the SP5 confocal microscope (Leica Microsystems). Average immunopositivity was measured in LAS AF software (LAS AF Lite version 2.6.3 8173, Leica Microsystems).

### 4.5. Determination of Activity of Antioxidative Enzymes in the Small Intestine

To investigate the antioxidative defence system, the small intestine was dissected and thoroughly rinsed with saline solution to remove traces of blood. A homogenate of the small intestine, prepared in sucrose buffer (0.25 M sucrose, 0.1 mM EDTA, and 50 mM Tris-HCl, pH 7.4), was used to measure the activities of the antioxidant enzymes. GPX activity was determined spectrophotometrically using t-butyl hydroperoxide as substrate [89] and expressed in nmol reduced NADPH/min/g protein. GST activity was measured according to the method of Habig et al. and expressed in nmol GSH/min/g protein [90]. Activity of GR was determined according to the method of Glatzle et al. and expressed in nmol NADPH/min/g protein [91]. Total SOD activity was determined according to the method described by Misra and Fridovich [92], but at 26 °C, and expressed in U/mg protein. Total specific SOD activity and MnSOD activity after inhibition with 4 mM potassium cyanide (KCN) were measured, and then CuZnSOD activity was calculated. SOD units were defined as the amount of enzyme inhibiting epinephrine oxidation by 50% under the appropriate reaction conditions. CAT was assayed according to Beutler [93], and the activity was expressed in μmol H_2_O_2_/min/g protein.

### 4.6. SDS-Polyacrylamide Gel Electrophoresis and Western Blot Analysis

To examine protein expression in the small intestine, a small intestine tissue homogenate was prepared in sucrose buffer containing protease and phosphatase inhibitors (Protease Inhibitor Mix HP, 39106.02, Phosphatase-Inhibitor-Mix I, 39050.02; SERVA Electrophoresis GmbH, Heidelberg, Germany), as described previously [86]. The homogenates were centrifuged at 37,500 rpm for 1.30 h at +4 °C. Prior to Western blotting, eight homogenates from each group were pooled in sets of four, yielding two samples per group, with each sample comprising homogenates from four animals. These homogenates were then used to determine protein concentration according to the method of Lowry et al. [94]. Twenty micrograms of each pooled homogenate was loaded onto a 10% or 12% SDS-polyacrylamide gel electrophoresis (PAGE) in independent lanes, yielding exactly two independent lanes per experimental group. The separated proteins were transferred to polyvinylidene fluoride (PVDF) membranes (10600023, Amersham Hybond P 0.45 PVDF, GE Healthcare Life Sciences, Sunderland, UK) overnight at +4 °C. Before immunoblotting, all membranes were incubated in 5% BSA dissolved in TBST (0.2% Tween 20, 50 mM Tris–HCl pH 7.6, 150 mM NaCl). Primary antibodies—β-tubulin (FNab09105, 1:10,000), anti-GLUT2 (ab192599, 1:1000), anti-JAM-1 (ab91501, 1:1000), anti-P2X7 (sc514962, 1:2000), anti-Bax (#2772, 1:1000) and anti-Bcl-2 (#2876, 1:1000)—were incubated overnight at 4 °C or for 1–2 h at room temperature with constant gentle rocking. Membranes were then washed six times for 5 min in TBST, after which HRP-conjugated anti-rabbit IgG secondary antibodies (ab205718, 1:20,000, Abcam) or horse anti-mouse (7076S, 1:1000, Cell Signaling Technology) were applied for 1 h at room temperature. Protein bands were visualised by chemiluminescence using luminol and 30% H_2_O_2_ and detected with an iBright CL1500 Imaging System (Thermo Fisher Scientific). Membranes were reprobed according to the stripping protocol, which involved fast rocking in 200 mM NaOH (2 × 5 min), followed by incubation in 5% BSA and reprobing with the appropriate primary antibodies. Quantitative analysis of immunoreactive bands was performed densitometrically using ImageJ software (National Institutes of Health, Bethesda, MD, USA). The ratio of the pixel number of the target protein band to the loading control was presented relative to the control group of young maturing rats, which was standardised as 100%.

### 4.7. Statistical Analyses

Statistical analyses were performed using GraphPad Prism version 9.5.1 (GraphPad Software, San Diego, CA, USA). Data are expressed as mean ± SEM. Data were analysed by independent-measures two-way ANOVA, followed by Tukey’s post hoc test to control the Type I error rate. For histomorphometric and immunohistochemical analyses, individual animal means (derived from 10 technical fields per specimen) were used as the biological replicate (n = 3/group) to avoid pseudoreplication. Western blot densitometric lanes were used solely for descriptive evaluation and were excluded from inferential statistical models because of sample pooling constraints. Only IP-GTT data were analysed by two-way repeated-measures ANOVA with Tukey’s post hoc test; Greenhouse–Geisser corrections were automatically applied if the assumption of sphericity was violated. Significance was set at *p* < 0.05.

## 5. Conclusions

Our study shows that the small intestine responds differently to a chronic, high-dose dextrose drinking regimen depending on biological maturity. The most pronounced alterations occurred in middle-aged rats receiving the 60% dextrose solution, characterised by impaired glucose handling, disrupted cell proliferation, compromised barrier function, and localised cellular damage. Furthermore, our findings indicate a parallel trend between tissue iron accumulation, localised inflammation, and weakened antioxidant defences, suggesting potential crosstalk among the HMGB1, NF-κB p65, and Nrf2 pathways under chronic dextrose overload. However, since this study relied entirely on tracking the localised distribution and correlation of these tissue markers, rather than using functional knockouts or specific pathway inhibitors, these parallel molecular changes should be interpreted cautiously as associative observations rather than as definitive evidence of a causally linked regulatory circuit. Although stable fasting HOMA-IR values indicate basal metabolic compensation in both age groups, dynamic morphological and molecular analyses show that chronic liquid dextrose intake induces localised small-intestinal stress that worsens with biological maturity. Elucidating these maturity-dependent tissue responses offers deeper insight into the potential health risks associated with excessive soft drink consumption and unregulated sports supplementation throughout the lifespan.

## Figures and Tables

**Table 1:** Effect of a high-dextrose drinking regimen on physiological, metabolic, and consumption parameters in young maturing and middle-aged adult rats.

	YOUNG-MATURING			MIDDLE-AGED			Two-Way ANOVA		
	Ctrl	20D	60D	Ctrl	20D	60D	Age	Dex	Interaction
Initial body weight (g)	67.0 ± 4.9	88.6 ± 1.8	82.4 ± 4.8	524.6 ± 27.6	533.2 ± 11.1	531.6 ± 16.1	<0.001	NS	NS
Final body weight (g)	307.4 ± 7.4	295.5 ± 8.7	312.0 ± 12.2	526.0 ± 28.5	561.7 ± 11.9	579.0 ± 24.2	<0.001	NS	NS
Weight gain (g)	240.4 ± 6.5	206.9 ± 9.7 *	229.6 ± 12.9	1.4 ± 1.7	28.5 ± 2.8 *	47.4 ± 8.9 ***	<0.001	<0.05	<0.001
Food intake (g/day/animal)	20.5 ± 1.5	12.9 ± 0.7 **	15.5 ± 0.8 *	24.8 ± 0.9	12.7 ± 0.8 ***	13.1 ± 0.9 ***	NS	<0.001	<0.01
Liquid intake (ml/day/animal)	33.0 ± 2.1	44.8 ± 3.6	31.9 ± 2.2	32.4 ± 1.2	80.3 ± 9.9 ***,▪,##	52.4 ± 7.1	<0.01	<0.001	<0.05
Caloric intake (kJ/day/animal)	288.0 ± 21.1	309.7 ± 16.7	313.4 ± 18.3	348.9 ± 13.1	402.7 ± 32.3	420.8 ± 61.2	<0.01	NS	NS
Dextrose intake (g/kg/day)	/	47.9 ± 11.2	34.6 ± 10.7	/	28.7 ± 2.9	26.2 ± 3.3	NS	NS	NS
Caloric intake from dextrose (%)	/	41.1 ± 1.8	30.3 ± 2.9	/	56.1 ± 2.8 #	54.7 ± 3.7 ##	<0.001	NS	NS
Glucose (mmol/L)	4.4 ± 0.2	4.3 ± 0.3	4.3 ± 0.2	4.7 ± 0.2	5.1 ± 0.2	5.1 ± 0.2	<0.001	NS	NS
Insulin (μU/mL)	30.4 ± 7.0	38.9 ± 2.9	30.0 ± 4.6	29.6 ± 0.9	24.2 ± 2.1	28.3 ± 1.4	NS	NS	NS
HOMA-IR	0.992 ± 0.49	1.241 ± 0.62	0.956 ± 0.47	1.031 ± 0.51	0.915 ± 0.45	1.067 ± 0.53	NS	NS	NS

Values are expressed as mean ± SEM. Statistical evaluations for body weights, glucose, insulin, and HOMA-IR were performed using independent-measures two-way ANOVA (n = 9 independent biological replicates per group). Daily food, liquid, and caloric consumption profiles, including relative dextrose parameters, were analyzed at the cage level (n = 3 to 4 independent experimental units per group) using values normalized by housing density (3 young maturing vs. 2–3 middle-aged rats per cage) to prevent pseudoreplication. Two-way ANOVA columns report the main statistical effects of age (representing maturity stages), dextrose drinking regimen (Dex), and their interaction. Non-significant differences are designated as NS. Comparison of treated groups with the corresponding control group (*); comparison of rats at different stages of maturity under the same dextrose drinking conditions (#); comparison of rats at the same maturity stage under different dextrose drinking regimens (▪). Statistical significance was set at *p* < 0.05, with *, #, ▪ *p* < 0.05; **, ## *p* < 0.01; *** *p* < 0.001.

**Table 2:** Morphometric parameters of the small intestinal compartments.

	YOUNG-MATURING			MIDDLE-AGED			Two-Way ANOVA		
	Ctrl	20D	60D	Ctrl	20D	60D	Age	Dex	Interaction
Villus height (µm)	457.6 ± 40.0	391.9 ± 38.0	327.0 ± 30.0	305.1 ± 30.0	317.2 ± 29.5	309.5 ± 29.0	<0.05	NS	NS
Crypt depth (µm)	112.0 ± 12.0	90.8 ± 8.8	86.0 ± 8.1	82.3 ± 7.6	78.1 ± 7.2	76.3 ± 6.5	<0.05	NS	NS
Villus width (µm)	78.9 ± 7.5	66.9 ± 6.0	82.6 ± 8.0	65.9 ± 6.1	72.7 ± 6.8	79.1 ± 7.7	NS	NS	NS
VSA (mm^2^)	0.11 ± 0.01	0.08 ± 0.01	0.08 ± 0.01	0.06 ± 0.01 ##	0.07 ± 0.01	0.08 ± 0.01	<0.01	NS	<0.05
Muscularis thickness (µm)	59.9 ± 4.0	62.2 ± 4.8	63.4 ± 6.4	47.8 ± 6.0	59.6 ± 5.2	40.8 ± 3.9	<0.05	NS	NS

Values are expressed as mean ± SEM (n = 3/group). Statistical evaluations were performed using independent-measures two-way ANOVA followed by Tukey’s post hoc test (n = 3 independent biological replicates per group, with each value derived from 10 non-overlapping technical microscopic fields per animal). Comparison of rats at different stages of maturity under the same dextrose drinking conditions (#). Statistical significance was set at *p* < 0.05, with ## *p* < 0.01.

## Data Availability

The original contributions presented in this study are included in the article/Supplementary Materials. Further inquiries can be directed to the corresponding author.

## Supplementary Materials

The following supporting information can be downloaded at https://www.mdpi.com/article/10.3390/ijms27167176/s1.
