# Supplementary material for: Effects of High-Dextrose Intake on the Structural and Functional Properties of the Small Intestine in Young-Maturing and Middle-Aged Rats
Source: Int J Mol Sci. 2026 Aug 11;27(16):7176. doi: 10.3390/ijms27167176 (PMC13513813; doi:10.3390/ijms27167176)

Supplementary Information

Effects of High-Dextrose Intake on the Structural and Functional Properties of the Small Intestine in Young-Maturing and Middle-Aged Rats

Anđelija Gudelj [1], Ilijana Grigorov [1,*], Vesna Otašević [1], Nevena Savić [1], Milica Markelić [2], Ana Stančić [1], Nikola Dajić [1] and Ksenija Veličković [2]

[1] Department of Molecular Biology, Institute for Biological Research "Siniša Stanković", National Institute of Republic of Serbia, University of Belgrade, 11000 Belgrade, Serbia
[2] Department of Cell and Tissue Biology, Faculty of Biology, University of Belgrade, 11000 Belgrade, Serbia
* Correspondence: iligri@ibiss.bg.ac.rs

Table S1: Detailed overview of experimental units, endpoind category and statistical model

Assay/ Parameter	Endpoint Category	Experimental Unit Entered Into Statistic	Final Statistical n Value (per group)	Data Presentation Format	A Follow-up Statistical Tool Used After Two-way ANOVA
**Body weight & Weight gain**	Primary outcome	Individual animal	n = 9	Mean ± SEM	Tukey's post-hoc test
**Food, Liquid & Calorie intake**	Primary outcome	Individual Cage (Normalized per animal)	n = 3/ young maturing<br>n = 4/ middle-aged adult	Mean ± SEM	Tukey's post-hoc test
**Dextrose intake & its % from calorie intake**	Primary outcome	Individual Cage (Normalized per animal)	n = 3/ young maturing<br>n = 4/ middle-aged adult	Mean ± SEM	Tukey's post-hoc test
**Glucose & Insulin**	Primary outcome	Individual animal	n = 9	Mean ± SEM	Tukey's post-hoc test
**IP-GTT, AUC & HOMA-IR**	Primary outcome	Individual animal	n = 9	Mean ± SEM	Tukey's post-hoc test
**Morphometric , IHC & IF analysis**	Primary outcome	Individual animal (Mean of 10 technical fields)	n = 3	Mean ± SEM	Tukey's post-hoc test
**Activity of Antioxidative Enzymes**	Secondary outcome	Individual animal	n = 9	Mean ± SEM	Tukey's post-hoc test
**Western blot Analysis**	Secondary outcome	Pooled samples	n = 2	Mean ± SEM	None<br>(Excluded from manuscript)

Figure S1: Evaluation of intestinal epithelial cell turnover via proliferation, apoptosis, and necrosis in young and middle-aged rats following high-dextrose drinking regimen.

Representative image of the small intestine from middle-aged adult rats treated with 60% dextrose, showing tissue injury, extensive lymphocyte infiltration, and numerous cells at various stages of apoptotic and necrotic cell death. The insert shows an enlarged area. During apoptosis, cells shrink and detach from neighbouring cells and the extracellular matrix, creating hollow spaces around the cells. Chromatin condenses into dense, compact masses near the nuclear envelope (nuclear heterochromatization; rectangle), resulting in the formation of pyknotic nuclei (black arrow) and, subsequently, apoptotic bodies (white arrow). In contrast, necrotic cells appear pale due to enzymatic protein degradation and water influx. Ultimately, chromatin is completely degraded by nucleases, leaving a faded or absent nucleus (ellipse).

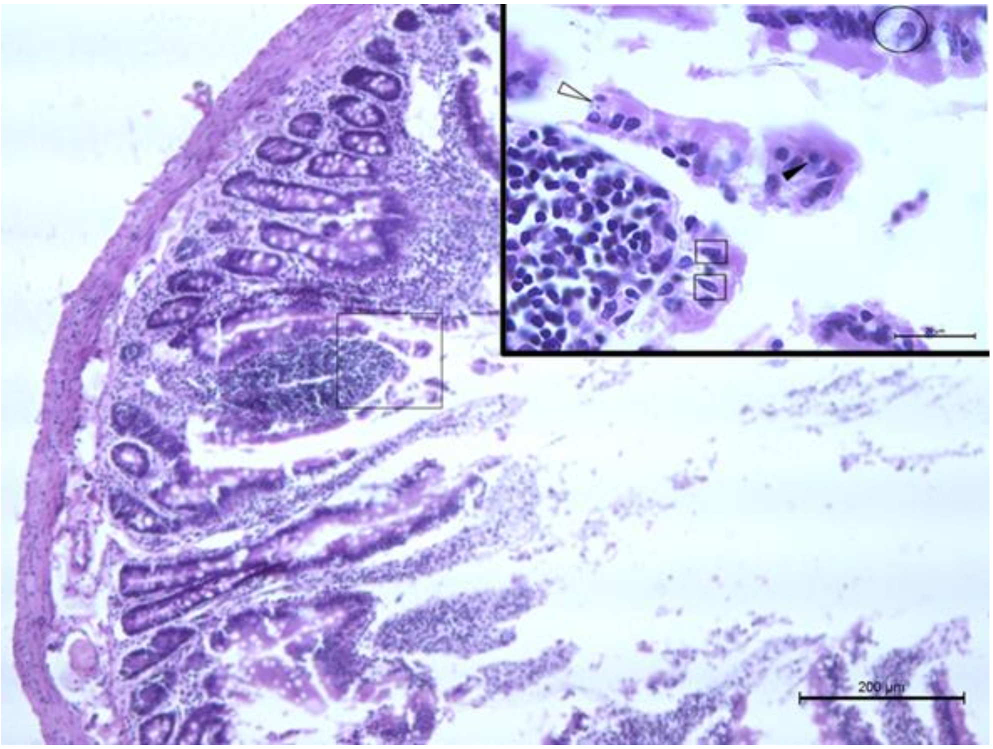

Original Western blots of whole homogenates from rat small intestine using anti-BAX and anti-Bcl-2 antibodies. Membranes represent original Western blots from main Figure 3E.

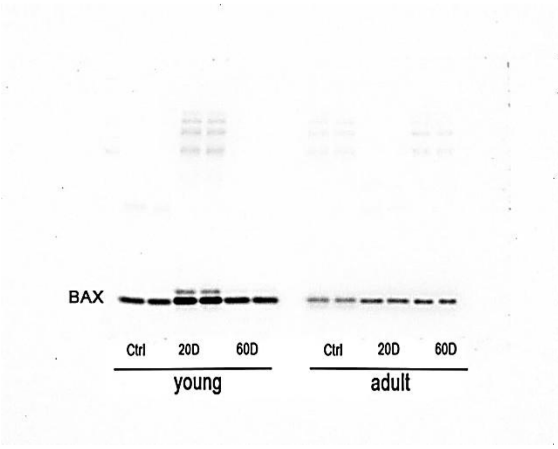

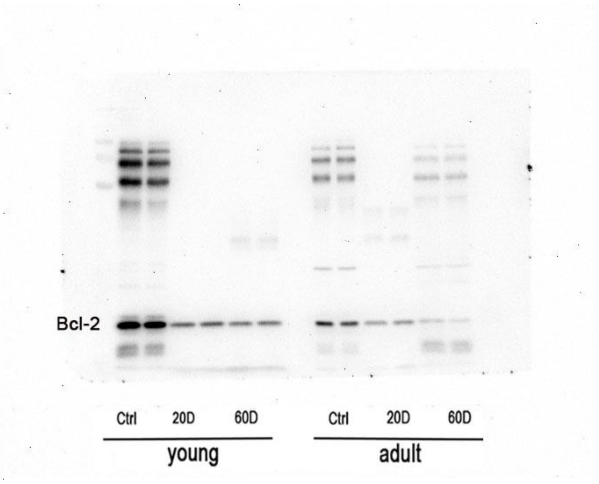

Figure S2: Assessment of junctional adhesion molecule 1(JAM-1), serotonin, and neuron-specific enolase (NSE) expression in the small intestine of young and middle-aged adult rats following high-dextrose drinking regimen: Original Western blots of whole homogenates of rat small intestine using anti-JAM-1 antibody.

Prior to Western blotting eight homogenates from each group (untreated (Ctrl); 20% dextrose-treated (20D); and 60% dextrose-treated (60D)) were pooled in sets of four, yielding two samples per group, with each sample comprising homogenates from four animals. Membrane 1 represents original Western blot of JAM-1 from main Figure 4A

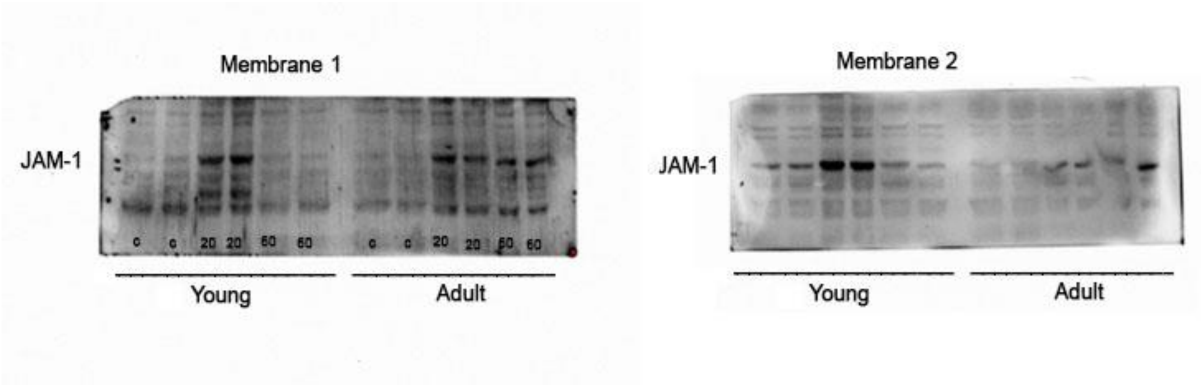

Figure S3. Assessment of glucose transporter 2 (GLUT2), P2X7 receptor, and ATP synthase expression and localization in the small intestine of young and middle-aged adult rats following high-dextrose drinking regimen:

Original Western blots of whole homogenates from rat small intestine using anti-GLUT2 and anti-P2X7 antibodies. Membranes 1 represent original Western blot of GLUT2 or P2X7 from main Figure 5A

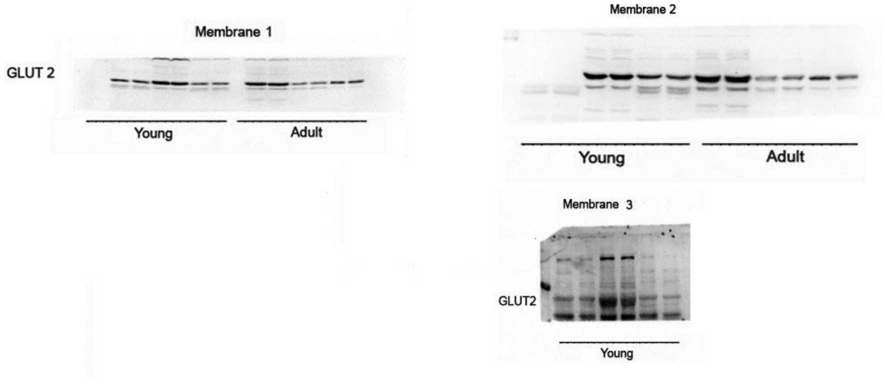

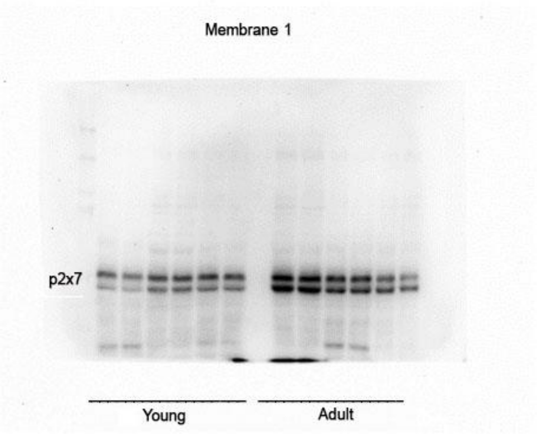

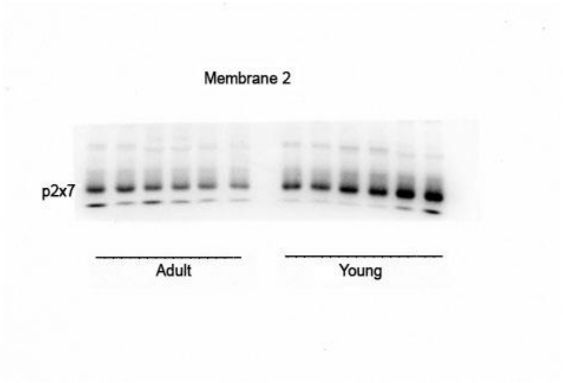

β TUBULIN 1. Original Western blot of β tubulin from main Figure 4A, JAM-1

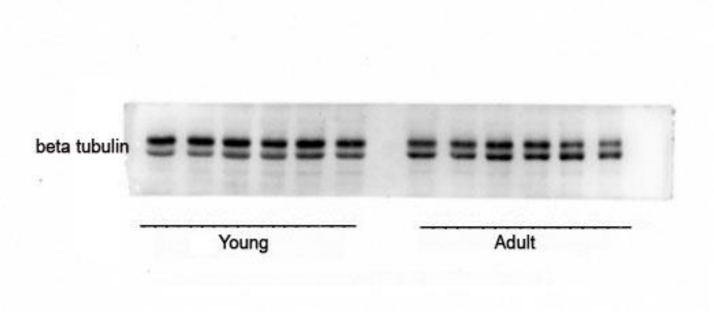

β TUBULIN 2. Original Western blot of β tubulin from main Figure 5A, GLUT2 and P2X7

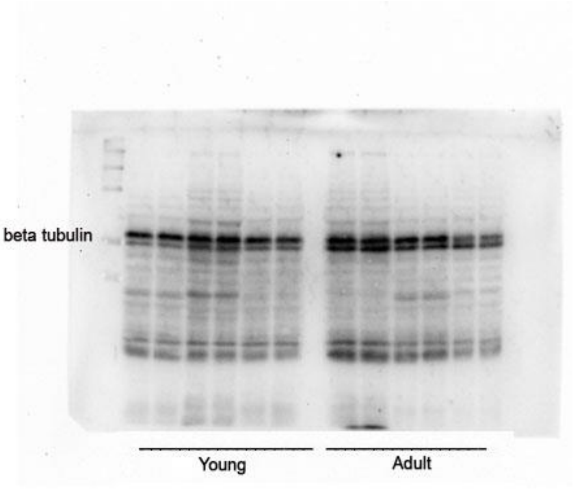

Supplement: Supplementary file 1 [file ijms-27-07176-s001.zip › ijms-4427465-supplementary.pdf]
